# Global identification of conserved post-transcriptional regulatory programs in trypanosomatids

**DOI:** 10.1093/nar/gkt647

**Published:** 2013-07-22

**Authors:** Hamed S. Najafabadi, Zhiquan Lu, Chad MacPherson, Vaibhav Mehta, Véronique Adoue, Tomi Pastinen, Reza Salavati

**Affiliations:** ^1^Institute of Parasitology, McGill University, 21111 Lakeshore Road, Ste. Anne de Bellevue, Montreal, Quebec H9X 3V9, Canada, ^2^McGill Centre for Bioinformatics, McGill University, 3649 Promenade Sir William Osler, Montreal, Quebec H3G 0B1, Canada, ^3^Department of Human Genetics, McGill University Health Centre, Montréal, Québec, Canada, ^4^McGill University and Genome Québec Innovation Centre, Montréal, Québec H3A 1A4, Canada, ^5^Department of Medical Genetics, McGill University Health Centre, Montréal, Québec, Canada and ^6^Department of Biochemistry, McGill University, McIntyre Medical Building, 3655 Promenade Sir William Osler, Montreal, Quebec H3G 1Y6, Canada

## Abstract

While regulatory programs are extensively studied at the level of transcription, elements that are involved in regulation of post-transcriptional processes are largely unknown, and methods for systematic identification of these elements are in early stages. Here, using a novel computational framework, we have integrated sequence information with several functional genomics data sets to characterize conserved regulatory programs of trypanosomatids, a group of eukaryotes that almost entirely rely on post-transcriptional processes for regulation of mRNA abundance. This analysis revealed a complex network of linear and structural RNA elements that potentially govern mRNA abundance across different life stages and environmental conditions. Furthermore, we show that the conserved regulatory network that we have identified is responsive to chemical perturbation of several biological functions in trypanosomatids. We have further characterized one of the most abundant regulatory RNA elements that we discovered, an AU-rich element (ARE) that can be found in 3′ untranslated region of many trypanosomatid genes. Using bioinformatics approaches as well as *in vitro* and *in vivo* experiments, we have identified three ELAV-like homologs, including the developmentally critical protein *Tb*RBP6, which regulate abundance of a large number of trypanosomatid ARE-containing transcripts. Together, these studies lay out a roadmap for characterization of mechanisms that modulate development and metabolic pathways in trypanosomatids.

## INTRODUCTION

Identification of the mechanisms that regulate cellular processes is crucial to understanding the cell development and behavior. Regulatory networks are considerably more complex in eukaryotes than in prokaryotes, consisting of myriads of regulatory interactions among proteins, RNA molecules and genomic DNA. Markedly, post-transcriptional events play a major role in the regulation of eukaryotic genes. While transcriptional regulation of gene expression has been the subject of many studies over the years (1,2), the widespread role of post-transcriptional events in gene regulation has rather recently come to light (3–6). Post-transcriptional regulation primarily involves the interaction of a *cis*-regulatory RNA element and a *trans*-acting element, which is usually either a microRNA or an RNA-binding protein.

Most *cis*-acting elements that regulate mRNA abundance at the post-transcriptional level are located in the mRNA untranslated region (UTR), many of which form distinct secondary structures that are specifically recognized by their *trans*-acting binding partners (7–9). Several studies have used computational methods to address the problem of identifying the structural RNA motifs that are involved in post-transcriptional regulation. These methods identify structural RNA regulatory elements based on commonality in a set of related sequences (10), the ability to explain expression data (11,12) or conservation across species (13–16). Many conservation-based methods require an aligned set of RNA sequences that contain the putative regulatory element(s) (17). These methods have limited applicability when the sequences are highly diverged and cannot be aligned reliably. In contrast, methods that identify structural RNA motifs from unaligned sequences assume that, in homologous sequences, similarity is limited to functional parts, such as regulatory elements (13). Therefore, these methods can identify structural motifs even within sets of sequences that cannot be aligned. Nonetheless, these methods consider only the conservation of *cis*-regulatory elements, discarding the useful information that the conservation of the regulatory ‘network’ provides.

‘Network-level’ conservation of regulatory programs implies that if A and B are two orthologous *trans*-acting regulatory elements in two different organisms, the target genes of A (i.e. the genes that are bound and regulated by A) are mostly the orthologs of the target genes of B. Combined with the notion that the binding preferences of *trans*-acting regulatory elements are conserved across species (18), network-level conservation has been successfully used to identify conserved *cis*-regulatory motifs (19,20), but has been so far limited to analysis of linear motifs in pairs of organisms.

Here, we introduce a general framework for identification of linear and structural motifs based on network-level conservation across multiple species. We use this framework to identify conserved post-transcriptional regulatory programs in trypanosomatids, a group of organisms in which gene regulation is mainly at the post-transcriptional level. Through extensive analysis of the expression landscape of trypanosomatid genes, we show the responsiveness of their regulatory network to developmental events as well as external and internal stimuli, indicating that trypanosomatid genes are modulated by a complex set of *cis*- and *trans*-regulatory elements at the post-transcriptional level within and across different life stages. Furthermore, we identify a highly abundant AU-rich element (ARE) that is potentially involved in developmental regulation of a large number of trypanosomatid transcripts through interaction with three remote homologs of ELAV-like proteins.

## MATERIALS AND METHODS

The methods that we used to identify and characterize the conserved post-transcriptional regulatory programs of trypanosomatids are briefly summarized here. The complete description of the materials and methods can be found in Supplementary Methods (Supplementary Methods, Supplementary Figures and Supplementary Tables are included within Supplementary Data File S1).

Supplementary Figure S1 schematically shows the statistical framework for identification of network-level conservation across multiple species, which is implemented in the software COSMOS (Conserved Structural Motif Search Tool, http://tinyurl.com/rCOSMOS). COSMOS examines a highly diverse library of ∼4.7 × 10^6^ possible linear and structural motifs to identify RNA elements whose pattern of presence and absence in the 3′UTRs of the genes is conserved across different organisms. COSMOS assumes, under the null hypothesis of no network-level conservation, a binomial distribution for the number of orthologous gene groups that have instances of a particular motif in their corresponding 3′UTRs in all the examined organisms. COSMOS identifies motifs that deviate from this binomial distribution and, therefore, show a significant conservation pattern (details can be found in the Supplementary Methods). We used COSMOS to identify RNA elements that are conserved across the genus of *Trypanosoma*, including *Trypanosoma brucei*, *Trypanosoma cruzi*, *Trypanosoma vivax*, *Trypanosoma congolense* (Supplementary Table S1).

Using various statistical measures, we used available microarray and RNA-Seq data (21–27) of *T. brucei* to validate the regulatory programs that COSMOS has identified. The rationale for this analysis was that functional instances of a biologically significant *cis*-regulatory element generally behave similarly in response to cell state. Therefore, transcripts that carry functional elements are often co-regulated in different conditions. We used the *T. brucei* expression data to identify elements that are specifically upregulated or downregulated in different life stages or genetic backgrounds [Mann–Whitney U test, Benjamini-corrected false discovery rate (FDR) ≤0.1], or elements that show a high degree of co-regulation across different conditions (Supplementary Table S2 and Supplementary Data Files S2–S4).

Furthermore, to analyze the responsiveness of the identified regulatory network to cell state and environmental stimuli, we treated procyclic form (PF) *T. brucei* cells with different chemicals, including ethidium bromide, Dimethyl sulfoxide (DMSO), HCl, NaOH, hygromycin, verapamil, G418, pentamidine, phleomycin and imidazole. The transcriptome of treated cells was analyzed on *T. brucei* microarrays from Pathogen Functional Genomics Resource Center, and up-/downregulation of mRNAs was measured in comparison with the transcriptome of nontreated cells. These data (Gene Expression Omnibus accession no. GSE37593, Supplementary Data File S5) were used to study the specific behavior of each conserved regulatory program in response to the chemical perturbations using Mann–Whitney U test of ranks.

Using PSI-BLAST, we identified three remote homologs of ELAV-like proteins that could potentially bind to a highly frequent ARE that was conserved across trypanosomatids and was validated by several independent expression data sets. We verified one of these binding partners using biochemical approaches. Briefly, we expressed a recombinant version of Tb927.3.2930 (*Tb*RBP6), and used gel-shift assays to examine the specific binding of this protein to the RNA element. We verified an additional binding partner of AREs using RNA-binding protein immunoprecipitation followed by high-throughput sequencing (RIP-Seq). Specifically, we created stable PF *T. brucei* cell lines expressing a tagged version of Tb927.8.6650, and used tandem affinity purification to pull-down this protein. The co-purified transcripts were analyzed by RNA-Seq (Gene Expression Omnibus accession no. GSE46160 and Supplementary Data File S6)

We validated the role of these two proteins along with an additional candidate protein, Tb927.7.5380, in regulating mRNA abundance of ARE-containing transcripts by inhibition or phenotypic activation of these proteins followed by microarray analysis of *T. brucei* transcriptome. Briefly, we created tetracycline-inducible stable PF cell lines harboring RNA interference (RNAi) constructs for the candidate proteins, and also stable cell lines overexpressing each of these proteins. The effect of each RNAi/overexpression on *T. brucei* transcriptome was studied using custom oligonucleotide microarrays from NimbleGen Inc. (Gene Expression Omnibus accession no. GSE46161 and Supplementary Data File S7).

## RESULTS AND DISCUSSION

### Identification of linear and structural regulatory motifs that are conserved at the network level

The statistical framework that we have developed allows us to measure the network-level conservation of potential regulatory motifs across multiple species (Supplementary Figure S1). Using this approach, we searched in a set of ∼4.7 × 10^6^ linear and structural motifs (see the Supplementary Methods) to identify 3′UTR regulatory elements that are conserved at the network level across different species of the genus of *Trypanosoma*, including *T. brucei*, *T. cruzi*, *T. congolense* and *T. vivax*. These organisms are known to regulate their genes post-transcriptionally, using *cis*-regulatory factors that are mainly located in the mRNA 3′UTRs (28–31).

**Figure 1.**
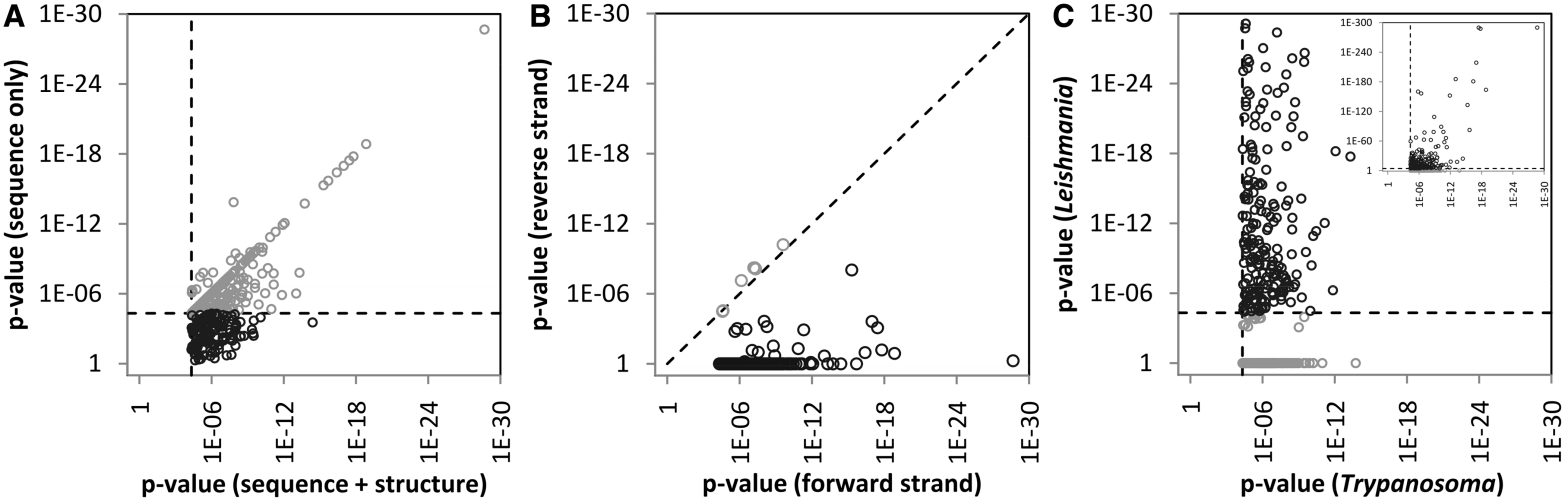
Network-level conservation identifies RNA motifs with structural and sequence information. We identified 388 linear and structural nonredundant motifs with significant network-level conservation across the genus of *Trypanosoma*. (**A**) When the structural information is discarded, 222 motifs remain significant for their network-level conservation (gray), while the conservation scores of 166 motifs drop below the significance threshold (black). The latter motifs are deemed to have indispensible structural information. (**B**) Almost all motifs are exclusively conserved in the forward strand, and not in the reverse strand. The few motifs whose *P*-values are similar in the forward and reverse strand (gray) contain palindromic sequences. (**C**) Of the 388 motifs that are conserved in the genus of *Trypanosoma*, 237 are also conserved in the genus of *Leishmania*, including *L. major*, *L. infantum*, *L. braziliensis* and *L. mexicana* (black). For visualization purposes, the main graph displays motifs whose *P*-values in *Leishmania* are greater than 10^−30^; the inset graph represents all motifs. Note the reverse order of *P*-values in the graphs, with smaller (significant) *P*-values toward the right/top of each chart.

After removal of redundant motifs, our search resulted in 388 putative regulatory elements that were highly conserved in the *Trypanosoma* genus with an estimated FDR of ∼0.01 (Supplementary Table S1). The structural information of 166 of these motifs is indispensable, meaning that if the structural information was discarded and only the sequence information was retained, these motifs would not be identified as conserved anymore (Figure 1A). Furthermore, as expected from RNA *cis*-regulatory elements, almost all of the identified motifs are only conserved on the forward strand of the DNA, and show little or no network-level conservation when the reverse strand is considered (Figure 1B). We also found that the motifs that we identified are conserved not only within the genus of *Trypanosoma*, but also within another branch of trypanosomatids, namely the genus of *Leishmania*, which includes parasitic species such as *Leishmania major*, *Leishmania infantum*, *Leishmania braziliensis* and *Leishmania mexicana* (Figure 1C). This suggests that these motifs have conserved their function beyond the *Trypanosoma* genus, and that the corresponding regulatory network is conserved at least across the order of *Trypanosomatida*. Also, potential functional interactions among several motifs of this network suggest that post-transcriptional regulatory programs in trypanosomatids might work cooperatively (Supplementary Figure S2), similar to the widespread functional interactions that has been observed among regulatory programs in different eukaryotes, particularly human (32).

Several of the motifs that we identified match already known *cis*-regulatory elements that are involved in post-transcriptional regulation of different transcripts in many organisms, including trypanosomatids. For example, the most conserved motif, CAUAGAN, matches the known binding site of the trypanosomatid cycling sequence binding proteins, which determine the stability of S phase-specific transcripts (33). Also, we were able to identify a structural motif that matches the well-studied histone 3′UTR stem-loop, consisting of a 6-bp stem and a four–nucleotide loop with the consensus sequence NGCUNUUNNNRNRGYN (the stem region is underlined). This motif is involved in transport and regulation of histone transcripts (34). As another example, we identified a highly conserved linear motif with the consensus sequence AUGUAN. This motif contains the core binding sequence of the PUF family of RNA-binding proteins (35). A similar motif has been previously reported to be overrepresented among several groups of co-regulated transcripts in trypanosomatids (36).

These few examples suggest that our statistical framework can successfully identify conserved regulatory elements that are involved in post-transcriptional gene regulation. To systematically validate the discovered motifs, we further analyzed them by examining their profiles across several microarray and RNA-Seq experiments, as described in the next section.

### The trypanosomatid post-transcriptional regulatory network defines RNA regulons

**Figure 2.**
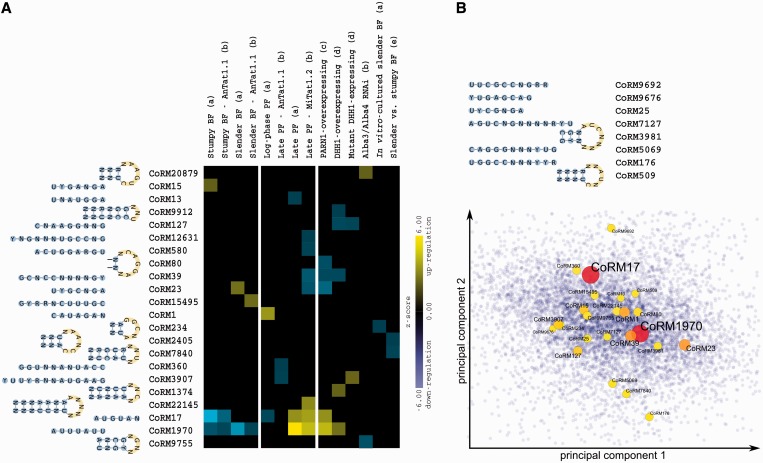
Conserved RNA motifs correlate with available microarray and RNA-Seq data of *T. brucei*. (**A**) Based on Mann–Whitney U test, 22 RNA motifs are significantly upregulated (yellow) or downregulated (blue) in at least one available expression data set of *T. brucei* (Benjamini-corrected FDR ≤ 0.1). The motif name along with the structure/sequence is shown on the left, with each column representing one expression data set. The letters in brackets correspond to the reference publications that describe each experiment: a: (23), b: (26), c: (25), d: (21), e: (22). (**B**) *Trypanosoma brucei* genes (blue dots) are mapped on the first two principal components of 22 previously published expression data sets (21–27). Local enrichment of motifs was examined in different regions of the expression hyperspace, with each region corresponding to the center of a set of co-regulated genes (see the ‘Materials and Methods’ section). Regions with significant local enrichment for motifs are highlighted in this figure by the circles. Larger/red circles represent higher enrichment z-scores. The sequences and structures of novel motifs that were validated by this analysis are shown on the top.

*Trypanosoma brucei* is one of the major disease-causing trypanosomatids, responsible for the deadly human African trypanosomiasis, also known as sleeping sickness. The life cycle of *T. brucei* mainly consists of the insect stage, dominated by PF parasites, and the mammalian stage, dominated by bloodstream form parasites. The transcriptome of *T. brucei* has been profiled through the life cycle as well as in different genetic backgrounds (21–27). By analyzing available microarray and RNA-Seq data of *T. brucei*, we found that several of the conserved RNA motifs that we have identified are potentially involved in regulation of genes through the life cycle of this organism. Specifically, 22 motifs show significant upregulation or downregulation in at least one experiment (Figure 2A).

In addition to analyzing individual microarray/RNA-Seq experiments, we analyzed the combination of expression profiles of *T. brucei* transcripts across different experiments to identify conserved motifs that occur in sets of co-regulated mRNAs. We identified 24 motifs that had significant enrichment among co-regulated genes of *T. brucei* (Figure 2B), eight of which could only be identified by this cross-experiment analysis and not by the enrichment analysis of single experiments. Overall, enrichment of the identified motifs among co-regulated genes suggests that the conserved post-transcriptional network of trypanosomatids determines RNA regulons, and is responsible for differential expression of genes across the life cycle and among different strains.

### The gene regulatory network of *T. brucei* is responsive to external stimuli and internal perturbations

**Figure 3.**
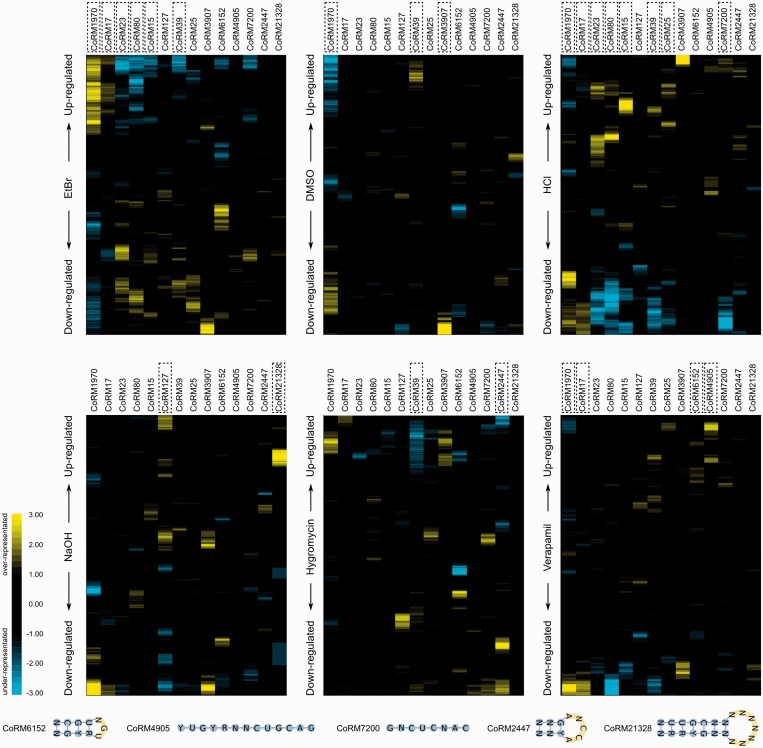
The GRN of *T. brucei* is responsive to environmental changes and stress conditions. Treatment of PF *T. brucei* cells with different chemicals results in significant up-/downregulation of several conserved RNA motifs. In each panel, genes that are upregulated are shown on the top, and downregulated genes on the bottom. Yellow indicates significant overrepresentation of motifs among genes with similar expression changes, and blue indicates significant underrepresentation of motifs. Over-/underrepresentation scores are calculated as the logarithm base 10 of cumulative *P*-values based on the Poisson distribution, shown here by the yellow–black–blue color gradient. Motifs that are significantly upregulated or downregulated in each experiment (Mann–Whitney U test, Benjamini-corrected FDR ≤ 0.1) are highlighted by the boxes. Five novel motifs were validated in this analysis, shown at the bottom. Ethidium bromide (EtBr) disrupts mitochondrial DNA replication and biogenesis in *T. brucei* (39); DMSO has a wide range of effects on cell permeability and molecular interactions; hygromycin inhibits polypeptide synthesis in eukaryotes and prokaryotes (40); and verapamil blocks calcium channels (41).

Early studies of *T. brucei* transcriptome suggested limited responsiveness of its gene regulatory network (GRN) to external and internal perturbations within the same life stage (37). Consequently, most studies of *T. brucei* transcriptome have focused on developmental gene regulation across different life stages of this parasite (22–24,26,28,38). Here, we examined the responsiveness of *T. brucei* transcriptome within the PF life stage by perturbing specific biological processes or imposing altered environmental conditions on the parasite. Specifically, we targeted mitochondrial DNA replication, protein synthesis and calcium ion transport, and created environmental stress conditions using several chemical compounds. Microarray analysis revealed widespread remodeling of *T. brucei* transcriptome in response to these perturbations (Supplementary Figure S3). Interestingly, we found that transcripts carrying our predicted motifs show specific and coordinated responses to the perturbations (Figure 3), suggesting critical roles for several of these motifs in sensing and adapting to stress conditions as well as regulating biological processes. The regulatory programs that responded to chemical perturbations included five potential regulatory elements that could not be validated using previously available microarray data.

### A high-confidence GRN suggests major regulatory role for AREs in *T.brucei*

We constructed a high-confidence GRN of *T. brucei* based on the assumption that the transcripts that carry functional instances of the same *cis*-regulatory motif must be co-regulated across different conditions. Therefore, the functional targets of a particular *trans*-regulatory element are transcripts that contain the corresponding *cis*-regulatory motif and are coexpressed with other carriers of that motif. This high-confidence GRN contains 1012 interactions between 917 genes and 12 regulatory elements (Supplementary Figure S4 and Supplementary File S2). CoRM17 (AUGUAN) and CoRM1970 (AUUUAUU) dominate the regulatory interactions of this GRN, targeting 508 and 328 genes, respectively. CoRM17 contains the core binding sequence of the PUF family of RNA-binding proteins (35), and expression data support a conserved role of PUF proteins in regulating CoRM17-containing transcripts (Supplementary Figure S5). On the other hand, CoRM1970 is an ARE that contains a core AUUUA sequence. AREs have been long known to be involved in regulation of mRNA stability in several eukaryotes (42). Different ARE-binding proteins have been characterized with a wide range of effects on target stability. While some ARE-binding proteins such as tristetraprolin, butyrate response factor 1 and AU-rich binding factor 1 destabilize their target transcripts (42), other proteins such as known examples of the ELAV-like family bind to AREs and stabilize the mRNA (43), mainly by protecting deadenylated transcripts against degradative enzymes (44). In the next sections, we examine the specific function of the AU-rich motif CoRM1970 in *T. brucei*.

#### An ARE with a central role in regulating mRNA stability

Congruent with the protecting function of known ELAV-like proteins, and based on analysis of previously reported microarray data (21), we found that CoRM1970-containing transcripts are protected from degradation in *T. brucei* cells that overexpress the DEAD-box RNA helicase DHH1 (Figure 2A); DHH1 is required for efficient decapping of deadenylated mRNAs, which is an essential step in deadenylation-dependent decay pathway (45). Also, we found that transcripts that have at least one instance of CoRM1970 are overrepresented in poly(A)^+^ mRNA content of *T. brucei* cells that overexpress poly(A)-specific ribonuclease 1 (25), suggesting that CoRM1970-containing transcripts are also protected against deadenylation activity of this enzyme (Figure 2A). This is in line with previous reports showing that ELAV-like proteins can simultaneously bind to the ARE and poly(A) tail (46), and thus possibly protect the poly(A) tail of ARE-containing transcripts.

These observations suggest the presence of homologs of ELAV-like proteins in *T. brucei*, with a central role in regulation of mRNA stability via interaction with CoRM1970 and protection of the mRNA against deadenylation and/or deadenylation-dependent decay. It has been previously shown that the expression of human HuR, a member of the ELAV-like protein family, in *T. brucei* results in stabilization of several ARE-containing mRNAs (47). However, the counterparts of HuR or any other ELAV-like proteins have not been characterized in *T. brucei*. To identify such counterparts, we performed PSI-BLAST analysis of all putative RNA-binding proteins of *T. brucei*, and found three potential remote homologs of ELAV-like proteins, including Tb927.3.2930, Tb927.8.6650 and Tb927.7.5380.

**Figure 4.**
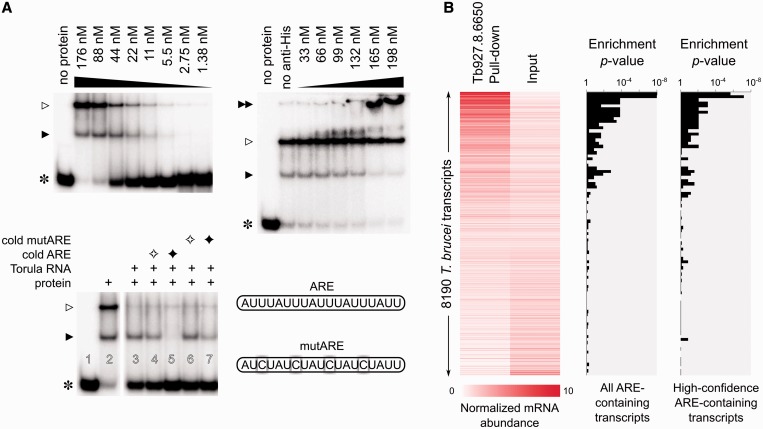
Validation of two candidate ELAV-like homologs as ARE-binding proteins. (**A**) Left-top: Recombinant *Tb*RBP6 binds in a concentration-dependent manner to an *in vitro*-transcribed ARE-containing RNA, as shown by gel-shift assays. The asterisk represents the input radiolabeled RNA, and the two arrowheads point to the RNA–protein complexes. The black arrowhead is the complex of the RNA with *Tb*RBP6, while the white arrowhead represents a (potentially misfolded) oligomeric form of *Tb*RBP6, as verified by mass spectrometry. The protein concentrations are indicated on top of the lanes. Right-top: Supershift assays with increasing amounts of anti-His antibody confirm that the ARE-binding protein is indeed the recombinant *Tb*RBP6. The anti-His antibody, *Tb*RBP6 and ARE-containing RNA form a complex (black double arrowhead) that is much heavier than the *Tb*RBP6–RNA complex (black arrowhead). The complex that contains the oligomeric *Tb*RBP6 (white arrowhead) does not show a supershift, possibly because the His-tags are buried inside. Left-bottom: Competition assays indicate that the interaction of monomeric *Tb*RBP6 with ARE-containing RNA is sequence-specific. The ARE-containing RNA does not detach from *Tb*RBP6 (black arrowhead) in the presence of Torula yeast total RNA (lane 3), or in the presence of up to 50-fold molar excess of cold competitor RNA that carries mutated versions of the AUUUAUU motif (lanes 6 and 7, the mutated substrate is shown on the right). Lanes 4 and 5 are competition positive controls, with cold wild-type ARE-containing RNA as the competitor RNA. White diamonds stand for cold competitor RNA (wild-type or mutated) in equal amounts as the radiolabeled ARE-containing RNA, and black diamonds stand for 50-fold excess of cold competitor RNA. (**B**) Tagged Tb927.8.6650 expressed from an ectopic allele in PF *T. brucei* co-purifies with ARE-containing transcripts. In the left panel, the profile of mRNA content co-purified with Tb927.8.6650 is compared with the total RNA (input) of *T. brucei*, with the abundance of the transcripts measured using high-throughput sequencing of the co-purified as well as the input RNA. ARE-containing transcripts as well as the high-confidence subset of ARE-mRNAs (Supplementary Figure S3 and Supplementary File S2) are enriched among the transcripts that specifically co-purify with Tb927.8.6650 (Fisher’s exact test on bins of 90 transcripts).

We first examined the binding specificity of Tb927.3.2930 (*Tb*RBP6). Although the amino acid sequence of *Tb*RBP6 shows only modest similarity to ELAV-like proteins (Supplementary Figure S6), we found that recombinant *Tb*RBP6 specifically interacts with RNA molecules that contain the CoRM1970 motif (Supplementary Figure S7 and Figure 4A). This interaction cannot be disrupted with Torula yeast total RNA, and is only slightly affected in the presence of large excess of competitor RNA containing mutated instances of CoRM1970, indicating that the interaction of *Tb*RBP6 with CoRM1970 is highly sequence-specific. We also examined the *in vivo* binding partners of another candidate, Tb927.8.6650, using RNA-binding protein immunoprecipitation followed by high-throughput sequencing (RIP-Seq). The results indicated that endogenous ARE-containing transcripts of *T. brucei* specifically co-purify with Tb927.8.6650 (Figure 4B), which is indicative of their interaction *in vivo*. We also found support from literature that the *T. brucei gambiense* protein Tbg972.7.6230, which is the homolog of our last candidate protein Tb927.7.5380, binds to an AU-rich motif that closely resembles CoRM1970 (Supplementary Figure S8). These data together provide *in vitro* and *in vivo* support that the three ELAV-like homologs that we have identified bind to AU-rich RNA sequences.

**Figure 5.**
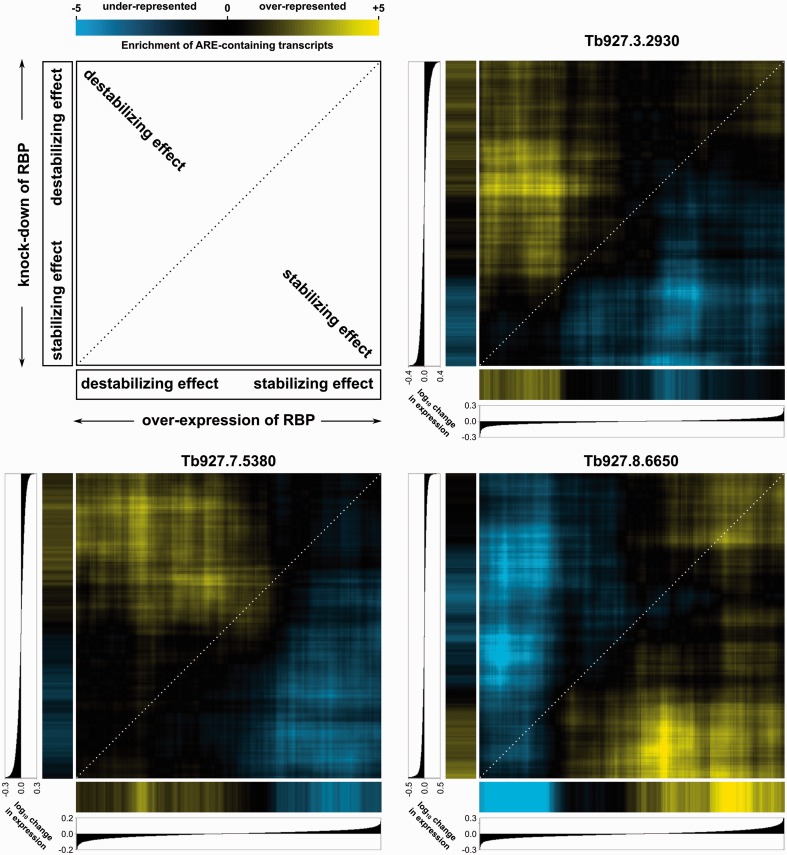
Inhibition and phenotypic activation of three ELAV-like homologs suggest roles in stabilizing and destabilizing ARE-mRNAs. Each protein was either phenotypically activated by tet-inducible overexpression or was inhibited by tet-inducible RNAi in PF *T. brucei* cells. Using oligonucleotide microarrays, the transcriptome after activation or inhibition was measured and compared with that of noninduced cells. On each axis, the transcripts are sorted by expression in tet-induced relative to control noninduced cells. Overrepresentation and underrepresentation of AREs are shown by yellow and blue patches, respectively, and are measured based on Fisher’s exact test on bins of 100 transcripts around each point in the 2-dimensional heatmap (see the color guide). Presence of yellow patches at the left-top part of each panel indicates a potential role of the protein in destabilizing AREs, and presence of yellow patches at the right-bottom part of each panel indicates role in stabilizing AREs.

We further functionally characterized the three ELAV-like homologs by inhibiting their expression using inducible RNAi, or by phenotypic activation of them in inducible stable cell lines that overexpress an ectopic allele for each protein, followed by microarray analysis of the transcriptome. Of the three candidates, inhibition and phenotypic activation of Tb927.8.6650 resulted in transcriptome remodeling expected of ELAV-like proteins: RNAi-mediated inhibition of Tb927.8.6650 downregulated ARE-containing transcripts, while phenotypic activation of Tb927.8.6650 upregulated ARE-mRNAs, indicating that Tb927.8.6650 stabilizes its ARE-containing targets (Figure 5). Interestingly, Tb927.3.2930 (*Tb*RBP6) and Tb927.7.5380 showed the inverse pattern, i.e. inhibition of them resulted in upregulation and phenotypic activation of them resulted in downregulation of ARE-containing transcripts (Figure 5), suggesting that these two proteins may play a potential role in destabilizing their ARE-containing targets. To our knowledge, these are the first examples of ELAV-like homologs that play a role in ARE-mRNA destabilization. Combined with recent studies showing that *Tb*RBP6 expression triggers differentiation of noninfective *T. brucei* cells to infective forms (48), these experiments suggest a central role of AREs in regulating trypanosomatid transcriptome and, subsequently, developmental events that lead to infectivity and disease.

### Final remarks

The statistical framework that we have introduced here provides a robust means for identification of regulatory programs that are conserved across multiple species. Most importantly, it provides an alignment-free method for identification of conserved *cis*-acting post-transcriptional regulatory motifs that contain sequence as well as structural information. This alignment-free framework allows identification of regulatory programs in genomes whose regulatory sequences have diverged extensively. Furthermore, in contrast to previous hypergeometric-based frameworks (20), our approach benefits from simultaneous analysis of multiple genomes, which leads to identification of motifs that are conserved in a large number of organisms.

We applied our framework to the genomes of trypanosomatids, a group of parasites with major health implications, in which regulatory mechanisms are poorly understood. Trypanosomatids provided a suitable benchmark for testing our framework, as gene regulation in these organisms is primarily post-transcriptional. Also, regulatory regions are poorly conserved in these organisms, meaning that conventional alignment-based approaches have limited applicability. Our approach was able to capture a large number of nonredundant *cis*-regulatory motifs, and we were able to validate 35 motifs using available expression data as well as expression data obtained from targeted perturbation studies (Supplementary Table S2). Interestingly, we found that several predicted motifs that are associated with transcriptome remodeling in *T. brucei* match *in vitro* determined binding sites of trypanosomatid RNA-binding proteins. Specifically, we examined the data from 10 RNAcompete assays of trypanosomatid RNA-binding proteins (18), and we found that the binding preferences of seven of these proteins have reciprocal best matches among the conserved trypanosomatid motifs (Supplementary Table S3). Of these seven matching motifs, six motifs were validated by expression analysis described above, indicating a significant enrichment of validated motifs among motifs that match *in vitro* binding data (9.5-fold enrichment, *P* < 3 × 10^−^^6^).

Our analysis provides the first global picture of post-transcriptional regulatory programs in trypanosomatids, and identifies major regulatory roles for several new candidate *cis*- and *trans*-regulatory elements, including previously unidentified homologs of ELAV-like proteins. Further characterization of these candidate regulatory elements will not only lead to a better understanding of the biology of these parasites and the diseases they create, but also may provide new targets for chemical therapeutics that affect and disrupt conserved key regulatory functions in these organisms.

## ACCESSION NUMBERS

GSE37593, GSE46160, GSE46161.

## SUPPLEMENTARY DATA

Supplementary Data are available at NAR Online.

## FUNDING

Natural Sciences and Engineering Research Council of Canada (NSERC) [328186 to R.S.]. Pathogen Functional Genomics Resource Center (PFGRC) provided *T. brucei* microarrays. Funding for open access charge: NSERC [328186].

*Conflict of interest statement*. None declared.

## Supplementary Material

Supplementary Data
