# Supplementary material for: Global identification of conserved post-transcriptional regulatory programs in trypanosomatids
Source: Nucleic Acids Res. 2013 Jul 22;41(18):8591–600. doi: 10.1093/nar/gkt647 (PMC3794602; doi:10.1093/nar/gkt647)
Supplement: Supplementary Data [file supp_41_18_8591__index.html]

Global identification of conserved post-transcriptional regulatory programs in trypanosomatids — Global identification of conserved post-transcriptional regulatory programs in trypanosomatids — Supplementary Data 

# Global identification of conserved post-transcriptional regulatory programs in trypanosomatids

## 

files

**Files in this Data Supplement:**

- Supplementary Data - zip file
