# Supplementary material for: Global identification of conserved post-transcriptional regulatory programs in trypanosomatids
Source: Nucleic Acids Res. 2013 Jul 22;41(18):8591–600. doi: 10.1093/nar/gkt647 (PMC3794602; doi:10.1093/nar/gkt647)
Supplement: Supplementary Data [file supp_gkt647_nar-01574-v-2013-File007.zip › NAR-01574-2013 Suppl files/Supplementary_Data_File_1.pdf]

Supplementary Materials

Global identification of conserved post-transcriptional regulatory programs in trypanosomatids

Hamed S. Najafabadi[1,2,¶,*], Zhiquan Lu[1,*], Chad MacPherson[1], Vaibhav Mehta[1], Véronique Adoue[3,4], Tomi Pastinen[3,4,5], Reza Salavati[1,2,6§]

[1] Institute of Parasitology, McGill University, 21111 Lakeshore Road, Ste. Anne de Bellevue, Montreal, Quebec H9X3V9, Canada

[2] McGill Centre for Bioinformatics, McGill University, 3649 Promenade Sir William Osler, Montreal, Quebec H3G0B1, Canada

[3] Department of Human Genetics, McGill University Health Centre, Montréal, Québec, Canada

[4] McGill University and Genome Québec Innovation Centre, Montréal, Québec, Canada

[5] Department of Medical Genetics, McGill University Health Centre, Montréal, Québec, Canada

[6] Department of Biochemistry, McGill University, McIntyre Medical Building, 3655 Promenade Sir William Osler, Montreal, Quebec H3G1Y6, Canada

[¶] Present address: The Donnelly Centre for Cellular and Biomolecular Research, University of Toronto, Toronto, Ontario M5S3E1, Canada

[§]Corresponding author: reza.salavati@mcgill.ca

*These authors contributed equally to this work.

Supplementary Methods

Trypanosomatid sequences and orthologs

Sequences of 3’ untranslated regions (3’ UTRs) for *Trypanosoma brucei*, *T. cruzi*, *T. vivax*, *T. congolense*, *Leishmania major*, *L. infantum*, *L. braziliensis*, and *L. mexicana* were downloaded from TriTrypDB v2.5 (1). We defined the 3’ UTR as the 1000-nucleotide region downstream of the stop codon, given that most 3’ UTR motifs are known to reside within this region. Although in some cases this region may also contain a part of the downstream coding sequence, it is presumed that such contaminating sequences do not have an effect on the analysis.
Orthologous genes were identified based on OrthoMCL v4 (2). Gene identifiers were converted to the most recent versions based on the list of gene aliases provided by TriTrypDB. Ortholog groups that contained several paralogs from the same organism were trimmed by randomly selecting one of the paralogs for each organism.

Identification of linear and structural motifs with significant network-level conservation

Supplementary Figure 1 schematically shows the statistical framework for identification of network-level conservation across multiple species, which is implemented in the software COSMOS (http://tinyurl.com/rCOSMOS). Consider *N* ortholog groups across *M* species with their associated regulatory sequences. We call an ortholog group a “keeper” of a motif if all *M* sequences that belong to this ortholog group have at least one instance of that motif. The number of these “keepers” determines the network-level conservation of the motif. COSMOS calculates the probability that a random distribution of the motif results in the observed number of keepers; small probability values indicate high conservation. The details of the calculations are as follows.
Given a particular motif and its instances in the regulatory regions of *N* genes in *M* species, the probability of occurrence of this motif in each species *i* is calculated as:

$$p_i = \frac{\sum_{1 \le j \le N} B\left(S_{i,j}\right)}{N},$$

where $B(S_{i,j})$ is 1 if the sequence that belongs to species *i* in the *j*th ortholog group has at least one instance of the motif; otherwise $B(S_{i,j})$ is 0. Thus, the probability of an ortholog group being a “keeper” under the null hypothesis of random distribution is:

$$p_{keeper} = \prod_{1 \le i \le M} p_i$$

The probability of observing at least *n* keepers is then calculated based on the binomial distribution:

$$p\left(K \ge n\right) = \sum_{n \le k \le N} f\left(k; N, p_{keeper}\right),$$

where *f* is the probability mass function of the binomial distribution, calculated as:

$$f(k;N,p)=\binom{N}{k}p^k(1-p)^{N-k}$$

COSMOS uses $p(K \geq n)$ as the conservation score for a motif, in which n is the number of keepers of that motif.

In this work, we considered ~4.7×10^6 linear and structural motifs, including all possible linear motifs with a maximum length of 7 nt and maximum number of 6 non-degenerate bases, and all possible stem-loop motifs with a maximum stem length of 8 bp, loop length of 3-7 nt, and maximum number of 6 non-degenerate bases. COMOS calculated the conservation scores as well as the false discovery rate-adjusted p-values (q-values). Motifs with q-values ≤0.01 were retained.

Identification and removal of redundant motifs

Consider the more conserved motif i and the less conserved motif j in the list of motifs that are sorted by ascending order of their conservation p-value ($1 \leq i < j$). Motif j is redundant given motif i if the instances of motif j in database D significantly overlap the instances of motif i. The overlap significance is calculated using the hypergeometric distribution as:

$$p\left(X \geq o_{i,j}\right)=\sum_{x=o_{i,j}}^{\min\left(O_i,n_j\right)} f\left(o_{i,j};N_j,O_i,n_j\right),$$

where f is the probability mass function of the hypergeometric distribution:

$$f\left(o_{i,j};N_j,O_i,n_j\right)=\frac{\binom{O_i}{o_{i,j}}\binom{N_j-O_i}{n_j-o_{i,j}}}{\binom{N_j}{n_j}}$$

N_j is the total number of sliding windows of length l_j in database D (l_j is the length of motif j), O_i is the number of sliding windows of length l_j that overlap at least one nucleotide of at least one instance of motif i, n_j is the total number of instances of motif j, $o_{i,j}$ is the number of instances of motif j that overlap at least one nucleotide of at least one instance of motif i. Motif j is considered redundant if there is at least one motif i ($i<j$) so that $p_{j|i} \leq \frac{0.01}{j-1}$. The denominator j is for Bonferroni correction of p-value, as each motif j is compared to j-1 motifs that are better conserved. In this study, we used the 3' UTRs of *T. brucei* genes as database D in order to identify redundant motifs.

Processing and merging available expression data

T. brucei microarray and RNA-seq data were obtained from multiple sources (3-9), and an expression compendium was compiled as follows. For E-MEXP-2025 and E-MEXP-2026 (3), the log ratio of induced vs. non-induced expression was calculated. For GSE18065 (6), the reported values were first

averaged separately for BF as well as for PF. Then, the average of BF and PF was calculated for each gene, resulting in a single average measurement for each gene. The log ratio of average BF to the overall average measurement and also the log ratio of average PF to the overall average measurement were then calculated. For GSE20593 (7), all reported log ratios from biological replicates were averaged. For GSE22571 (8), the following experiments were averaged, resulting in a single measurement for each gene: GSM560209, GSM560212, GSM560213, GSM560214. Then, the log ratio of each experiment to this overall average measurement was calculated. Also, the log ratio of GSM560208:GSM560207 and the log ratio of GSM560211:GSM560210 were calculated. For GSE24275 (9), the total log ratio was used. The rest of the data were obtained from the supplementary information of the corresponding articles. Gene identifiers were converted to the most recent version according to TriTrypDB, and the datasets were merged into a single expression compendium. Genes that were present in only a subset of the datasets were included in the expression compendium; thus, the compendium contains missing values. Each experiment (column) was then normalized to have an average of zero and standard deviation of one across different genes, as described before (10). It should be noted that this normalization does not affect single-array Mann-Whitney U-based analysis as described later, but is important for calculation of Pearson correlation coefficients across multiple experiments. The normalized matrix can be found in Supplementary Data File 3.

Leishmania microarray data were obtained from three previous publications (11-13). The following sets of experiments were averaged since they were biological replicates: S_1[GSM98805, GSM98806, GSM98870]; S_2[GSM99790, GSM99791, GSM99792]; S_3[GSM99795, GSM99796, GSM99797]; S_4[GSM99798, GSM99799]; S_5[GSM251641, GSM251642, GSM251643, GSM251644]; S_6[GSM251645, GSM251646, GSM251647, GSM251648]; S_7[GSM291427, GSM291428, GSM291429, GSM291430]. Also, the overall average values for experiments that belonged to the same GEO series were calculated, and the log ratios of replicate average to the series average were reported for each of the sets S_1 to S_7. The expression profiles were mapped to *T. brucei* genes based on homology, and datasets were merged and normalized as above (Supplementary Data File 4).

Chemical treatment of PF *T. brucei*

PF *T. brucei* cells were treated with different chemicals and drugs in order to perturb specific biological processes or create environmental stress conditions. Wild-type PF *T. brucei* cell line IsTat 1.7A (14) was grown in SDM-79 medium in 26 °C, while the cell count was kept between 1×10^7 and 3×10^7 cells/ml. The cells were treated with either 2.65 µg/ml ethidium bromide, 3.1% (v/v) DMSO, 0.31% (v/v) HCl, 12.5 mM NaOH, 1.13 µg/ml hygromycin, 1.9 µM verapamil, 1.13 µg/ml G418, 130 nM pentamidine, 2.5×10^{-3} % (v/v) Triton-X, 31 ng/ml of phleomycin, or 7.8 mM imidazole. The concentrations were chosen based on the EC_{50} values of these chemicals for inhibition of *T. brucei* growth, as determined by growing *T. brucei* cells in the presence of different concentrations of chemicals. We chose the EC_{50} in order to ensure that the target biological process of each chemical is affected at the selected concentration.

Microarray analysis of chemical perturbations

T. brucei cells were collected 48 h after treatment, and total RNA was extracted using TRIzol Reagent (Invitrogen) and was further purified using RNeasy Mini Kit (Qiagen) as per manufacturers' instructions. RNA quality was examined using Agilent 2100 Bioanalyzer prior to cDNA preparation. 25 μg RNA was incubated with 9 μg oligo(dT) primer ($dT_{23}VN$, where V is a mixture of A, C and G, and N is any nucleotide) at 70 °C for 10 min, and Cy5-labeled cDNA was synthesized using Superscript III Reverse Transcriptase (Invitrogen) in the presence of 10mM DTT, 0.5 mM of each of dATP, dGTP and dTTP, 0.05 mM dCTP, and 0.05 mM of Cy5-dCTP as per manufacturer's instructions. Control cDNA from untreated PF *T. brucei* cells was prepared similarly using Cy3-dCTP. RNA was hydrolyzed by RNase A and RNase H, and cDNA was cleaned up using Qiagen PCR purification kit. Equal amounts of Cy3/Cy5-labeled cDNA were mixed and hybridized to version 4 of *T. brucei* microarrays from Pathogen Functional Genomics Resource Center as described before (15). Microarrays were scanned using ScanArray Express (PerkinElmer) and the acquired images were quantified using ScanArray Express software with lowess normalization. The value for each probe was set to the binary logarithm of treated/control (Cy5/Cy3) median signal ratio, and different probes of each gene were averaged. Results of different chemical treatments were merged to obtain a matrix in which each row represented a gene and each column represented an experiment. This matrix is available via Gene Expression Omnibus (accession no. GSE37593). Each column was then normalized to have an average of 0.0 and standard deviation of 1.0. Then, each row was normalized to have an average of 0.0. The latter normalization was aimed to neutralize the expression changes that represented a general response to stress and cell death, retaining only the expression changes that represented the specific response of the cells to the corresponding chemical treatment. Gene identifiers were converted to the most recent TriTrypDB version. The normalized matrix can be found in Supplementary Data File 5.

Identification of up- or down-regulated motifs

In order to identify motifs whose corresponding transcripts were significantly up- or down-regulated in previously published expression profiles or in our microarray data, we used the standard Mann-Whitney U test. For each motif, we compared the distribution of values for motif-containing transcripts to the distribution of values for transcripts that lacked that motif. For each microarray/RNA-seq experiment, all transcripts were sorted in the descending order of their corresponding values, and for each motif the sum of ranks of the transcripts that contained at least one instance was calculated. The z-score was then calculated as:

$$z = \frac{m_R - R}{\sigma_R},$$

where R is the sum of ranks, m_R is the average expected R calculated as $[n(n+1) + n\times\acute{n}]/2$ (where n is the number of transcripts that have the motif and $\acute{n}$ is the number of transcripts that do not have any instance of the motif), and σ_R is the standard deviation of R, calculated as:

$$\sigma_R = \sqrt{\frac{n \cdot n'(n + n' + 1)}{12}}$$

Positive z-scores indicate up-regulation, and negative z-scores indicate down-regulation. False discovery rate (FDR) at different z-score cutoffs was determined by performing the same analysis on 100 randomly shuffled motif occurrence profiles, setting the cutoff at FDR ≤ 0.1.

Identification of motifs with local enrichment in the expression hyperspace

In order to identify the motifs that occurred in co-regulated transcripts across the previously published microarray/RNA-seq datasets (3-9), we searched for regions in the expression hyperspace where motifs were significantly enriched. Each point in this hyperspace was expressed as a vector of size 22, representing the 22 measurements in the datasets from previous publications. For each point in the hyperspace, genes were first sorted by the descending order of their Pearson correlation with the vector that corresponded to that point, and the local enrichment of each motif was measured using Mann-Whitney U test. Significant positive z-scores were determined by shuffling the motif occurrence profiles across the transcripts and recalculating the z-scores as described in the previous section (FDR$\leq$0.1). Using this method, we examined motif enrichment around 8035 points in the expression hyperspace, each point corresponding to a *T. brucei* gene. By adapting this approach, we limited our search to regions in the hyperspace that were populated by genes, rather than deserted regions. This approach also identified genes whose expression profiles were highly correlated with certain motifs; this information was used to construct the high-confidence GRN of *T. brucei*.

Gel-shift assays for interaction of Tb927.3.2930 (*Tb*RBP6) with CoRM1970

*Tb*RBP6 was amplified from 29-13 *T. brucei* genome (primers are listed in Supplementary Table 4), and was cloned between the *Bgl*II and *Xho*I sites of pET-30b vector for expression of His-tagged *Tb*RBP6 in Rosetta(DE3)pLysS *E. coli* (Novagen). The recombinant protein was purified using IMAC Ni-charged resin (Bio-Rad) as per manufacturer's instructions. After elution, the protein was concentrated using an Amicon filter unit (Millipore) with nominal molecular weight limit of 10 kDa, and was dialyzed with Tris buffer (20 mM Tris-Cl at pH 7.6, 50 mM KCl and 5 mM MgCl2). The protein concentration was measured on a quantitative Western blot using Versadoc (Bio-Rad).

Gel-shift assays were performed by incubating varying concentrations of the purified protein (0.345 nM – 176 nM) with 0.625 nM of [^{32}P] pCp 3' end-labeled ARE-containing RNA (Supplementary Figure 6) in RBB50 buffer (20 mM Tris-Cl at pH 7.6, 50 mM KCl, 5 mM $MgCl_2$, 100 µg/mL BSA, 10% glycerol and 1mM DTT) for 30 minutes at room temperature. The RNA-protein complex was then resolved on 10% TBE gel (Bio-Rad).

Competition experiments followed a similar procedure, except that a fixed concentration of the purified protein (66 nM) was used with 0.625 nM of labeled ARE-containing RNA, followed by addition of 3.5 µg of Torula yeast RNA (carrier). Then, unlabeled competitor RNA (Supplementary Figure 6) was added either at 1:1 molar ratio, or at 50-fold molar excess compared to the labeled RNA. Supershift experiments

were performed similarly, except that varying concentrations of anti-His monoclonal antibody (Clontech) were added to the reaction before resolving the complexes on 10% TBE gel.

RNA-binding protein immunoprecipitation followed by high-throughput sequencing (RIP-Seq) for identification of binding targets of Tb927.8.6650 (*Tb*DRBD13)

*Tb*DRBD13 was amplified from 29-13 *T. brucei* genome (primers are listed in Supplementary Table 4), and was cloned in pLew79-TAP between the *Hind*III and *Bam*HI sites of the plasmid. *T. brucei* procyclic-form (PF) 29-13 cells co-expressing the *tet* repressor and T7 RNA polymerase were grown in SDM-79 medium containing 10% fetal bovine serum and supplemented with 15 µg/ml G418 and 25 µg/ml hygromycin. Cells were transfected by electroporation with 10µg of Not1 linearized pLew79-DRBD13 plasmid, and selected in the presence of 2.5 µg/ml phleomycin. After stable cells were established, 4L of cells were induced to express TAP-tagged *Tb*DRBD13 by adding 100ng/ml tetracycline to the culture medium. Cells were harvested 48h after induction. Tandem affinity purification of *Tb*DRBD13 was performed as previously described (16). RNA associated with *Tb*DRBD13 was extracted from the calmodulin column eluate using TRIzol Reagent (Invitrogen), treated with DNase-free (Ambion), and further purified by phenol-chloroform extraction. This purified RNA was used to prepare libraries with standard Epicentre (Illumina) RNA-Seq Library Preparation kit. Protocol was adapted for the RIP-Seq sample: after phenol-chloroform extraction, RNA was directly eluted in the "Elute, Prime, Fragment Mix" from the kit (first steps skipped) and then followed standard procedure. Library quality was assessed by Agilent 2100 BioAnalyzer (Agilent Technologies). Samples were indexed and sequenced using Illumina HiSeq 2000 (100bp single-end). Total RNA from induced cells was analyzed in parallel, with the addition of a Ribo-zero depletion step for library preparation. Data are available via Gene Expression Omnibus (accession no. GSE46160).

Phenotypic activation and repression for functional characterization of Tb927.3.2930 (*Tb*RBP6), Tb927.7.5380 (*Tb*DRBD12), and Tb927.8.6650 (*Tb*DRBD13).

Target gene fragments for RNAi were selected based on the default settings of the RNAit software (17). They were amplified from *T. brucei* PF 29-13 genomic DNA and inserted into p2T7-177 between the *Bam*HI and *Hind*III sites of the plasmid. Constructs for inducible over-expression were generated by insertion of the coding sequence into pLew79-TAP between the *Hind*III and *Bam*HI sites of the plasmid. The primer pairs used to generate the DNA fragments for RNAi and over-expression constructs are summarized in Supplementary Table 4.
RNAi and over-expression were induced by adding 1 µg/ml and 100 ng/ml, respectively, of tetracycline to the culture medium. Cells were harvested after 48h, except for DRBD13 RNAi, which was harvested 24h after induction. Total RNA was extracted using TRIzol Reagent (Invitrogen) and further purified using RNeasy Mini Kit (Qiagen) according to the manufacturer's protocol. RNA was treated with DNase-free (Ambion) and synthesized into cDNA using TaqMan Reverse Transcription Reagents with random hexamer priming (Applied Biosystems). Labeling and hybridization was performed by NimbleGen

Systems Inc., Madison, WI USA, following their standard operating protocol. Non-induced cells were analyzed in parallel.

Supplementary Figures

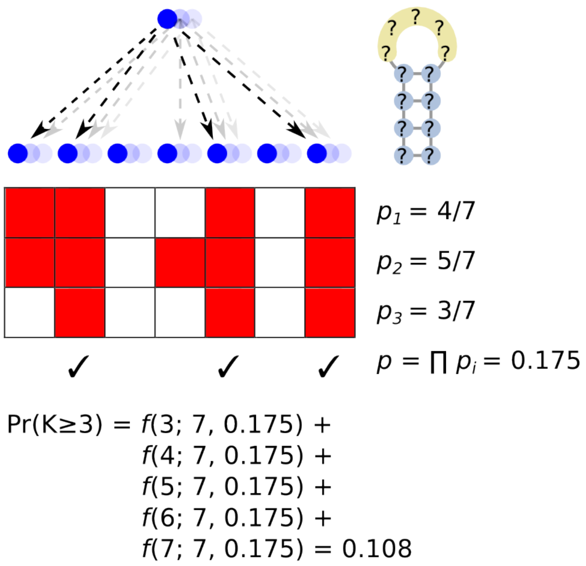

Supplementary Figure 1 – Finding motifs with high network-level conservation. COSMOS (Conserved Structural Motif Search tool) uses a binomial distribution-based statistical framework in order to identify motifs that are highly conserved at network level, i.e. motifs that correspond to the binding sites of conserved *trans*-regulatory factors with conserved regulatory networks (top of the figure). Given a particular motif with sequence/structure information, COSMOS estimates the probability of occurrence of the motif in a regulatory region in each organism (p_1, p_2 and p_3), and then uses them to predict the probability (p) of observing a "keeper" (shown by the check marks), i.e. an ortholog group in which all genes contain at least one instance of that motif in their regulatory region. Then, using the binomial distribution, COSMOS estimates the probability of the observed number of keepers under the null hypothesis of random motif distribution. Small probability values correspond to motifs that are highly conserved at network level.

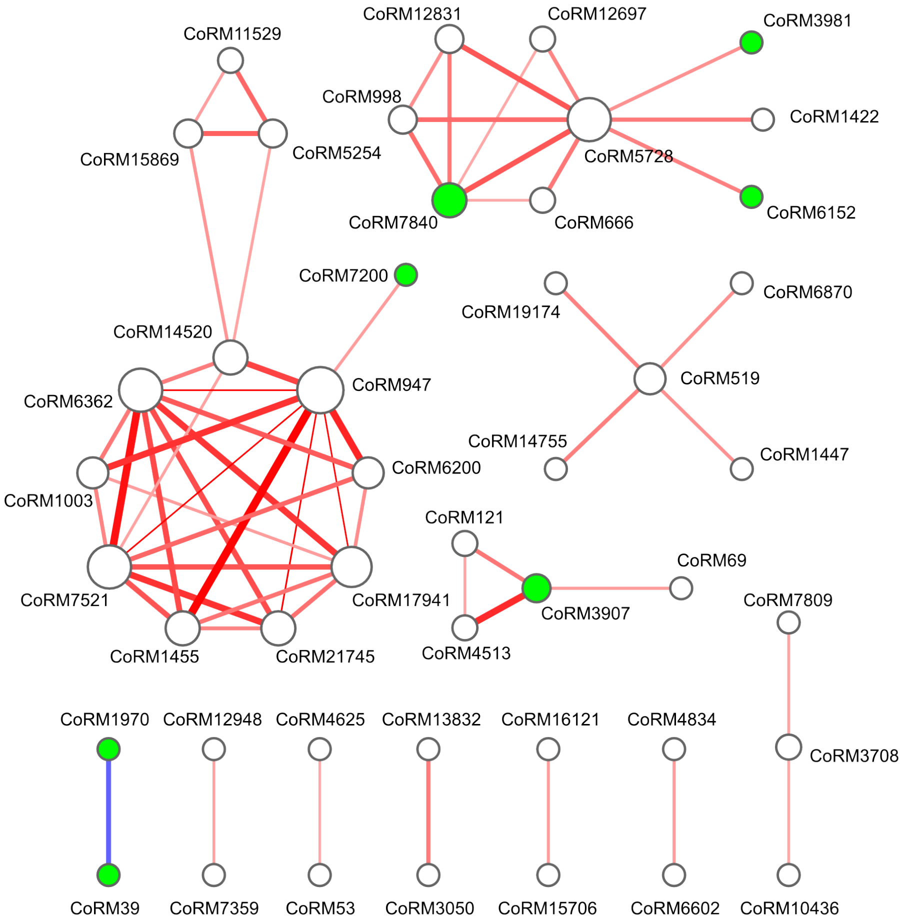

Supplementary Figure 2 – Regulatory interactions in *T. brucei*. Regulatory programs that act cooperatively usually target the same transcripts. For example, two RNA-binding proteins may bind to the same transcripts, resulting in a combined regulatory effect. To identify such cooperative interactions, we have searched for motifs that co-occur in transcripts more often than expected by chance. We identified 60 positive interactions and one negative interaction among 46 predicted conserved motifs in *T. brucei*. For each pair of motifs, functional interaction was identified based on the extent of overlap/exclusiveness of their carrier transcripts. Briefly, motif A is deemed to have a positive functional interaction with motif B if the target set of motif A (i.e. transcripts that harbor an instance of A in their 3' UTRs) significantly overlaps the target set of motif B. On the other hand, if the target set of motif A significantly excludes the target transcripts of motif B, A and B are deemed to have a potential negative interaction. This overlap/exclusion of target genes was measured by Fisher's exact test at Bonferroni-corrected *p*-value cutoff of 0.025. In this figure, red edges indicate positive interactions (significant overlap between the target sets of two motifs) and the blue edge represents negative interaction (an exclusive pair of motif). The overlap/exclusion *p*-value is shown by the thickness of the edges, with more significant interactions represented by thicker edges. The node size represents the number of functional interactions (degree) for each motif. Green nodes stand for motifs that were validated in this study based on previous expression data or based on microarray analysis of chemical perturbations.

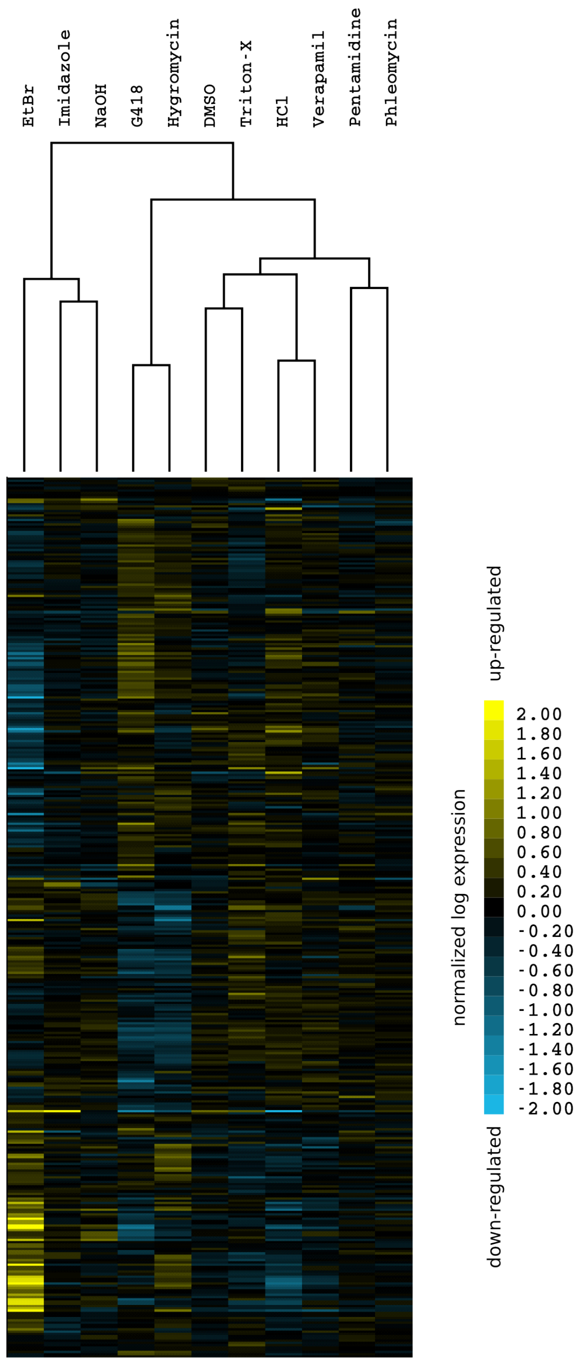

Supplementary Figure 3 – Expression profiling of chemical perturbations in *T. brucei*. Each row represents one gene, and each column one experiment. Columns are normalized to have an average of 0 and standard deviation of 1. Yellow and blue represent up- and down-regulation, respectively. For visualization purposes, genes with missing values are not shown here (638 transcripts out of 8299 transcripts had missing values). Missing values were because of low signal-to-noise ratio and, thus, unreliable measurements in all probes corresponding to a gene.

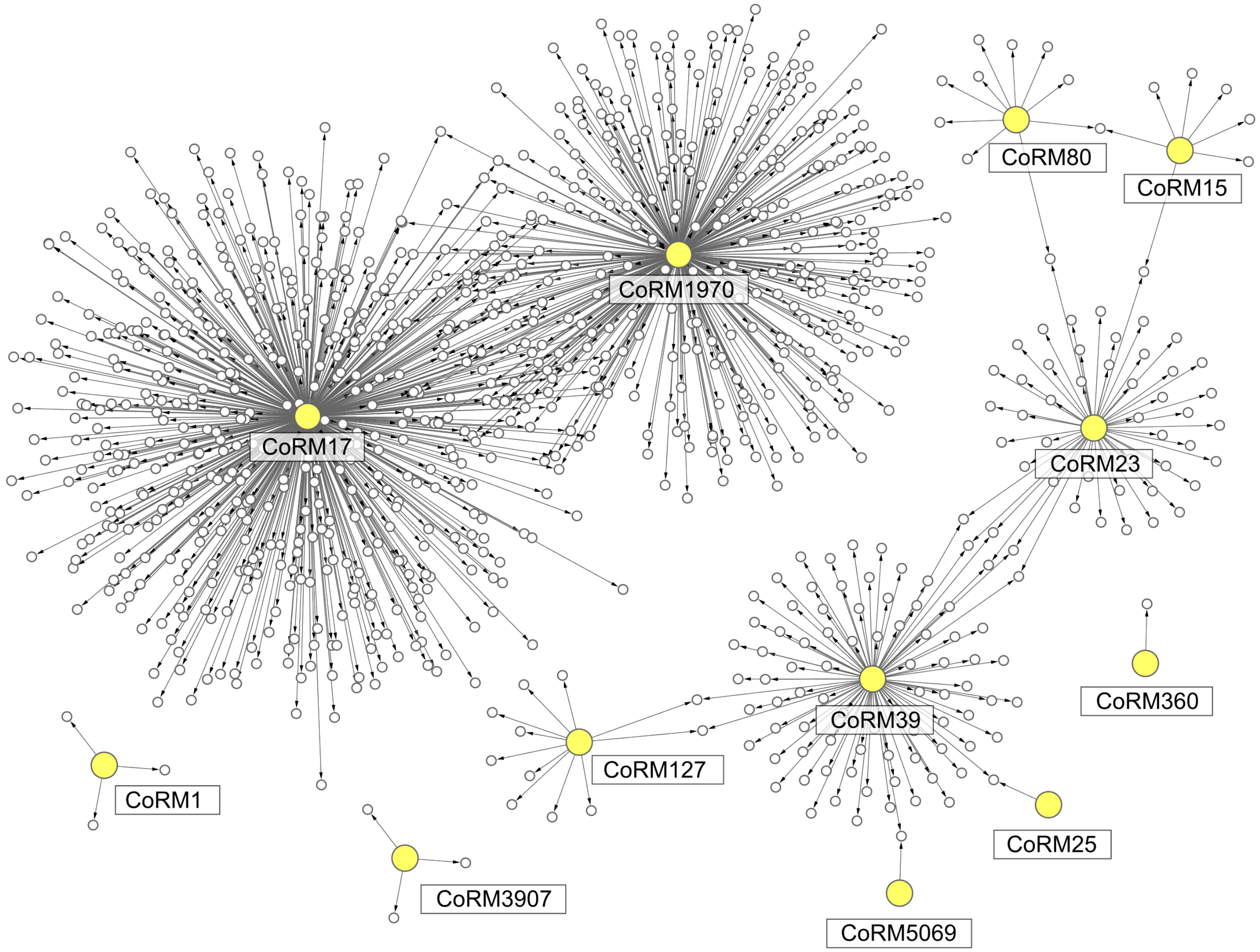

Supplementary Figure 4 – The high-confidence GRN of *T. brucei*. A gene is a high-confidence target of a regulatory element if it contains the corresponding motif in its 3' UTR, and if its expression pattern across previously published expression datasets (3-9) significantly correlates with the expression patterns of other transcripts that contain that motif. Target genes are shown by blank circle, and the arrows demonstrate regulatory relationships. Details can be found in Supplementary Data File 2. The number of high-confidence targets for each of the motifs is as follows: CoRM1: 3; CoRM15: 7; CoRM17: 508; CoRM23: 51; CoRM25: 1; CoRM39: 88; CoRM80: 10; CoRM127: 11; CoRM360: 1; CoRM1970: 328; CoRM3907: 3; CoRM5069: 1.

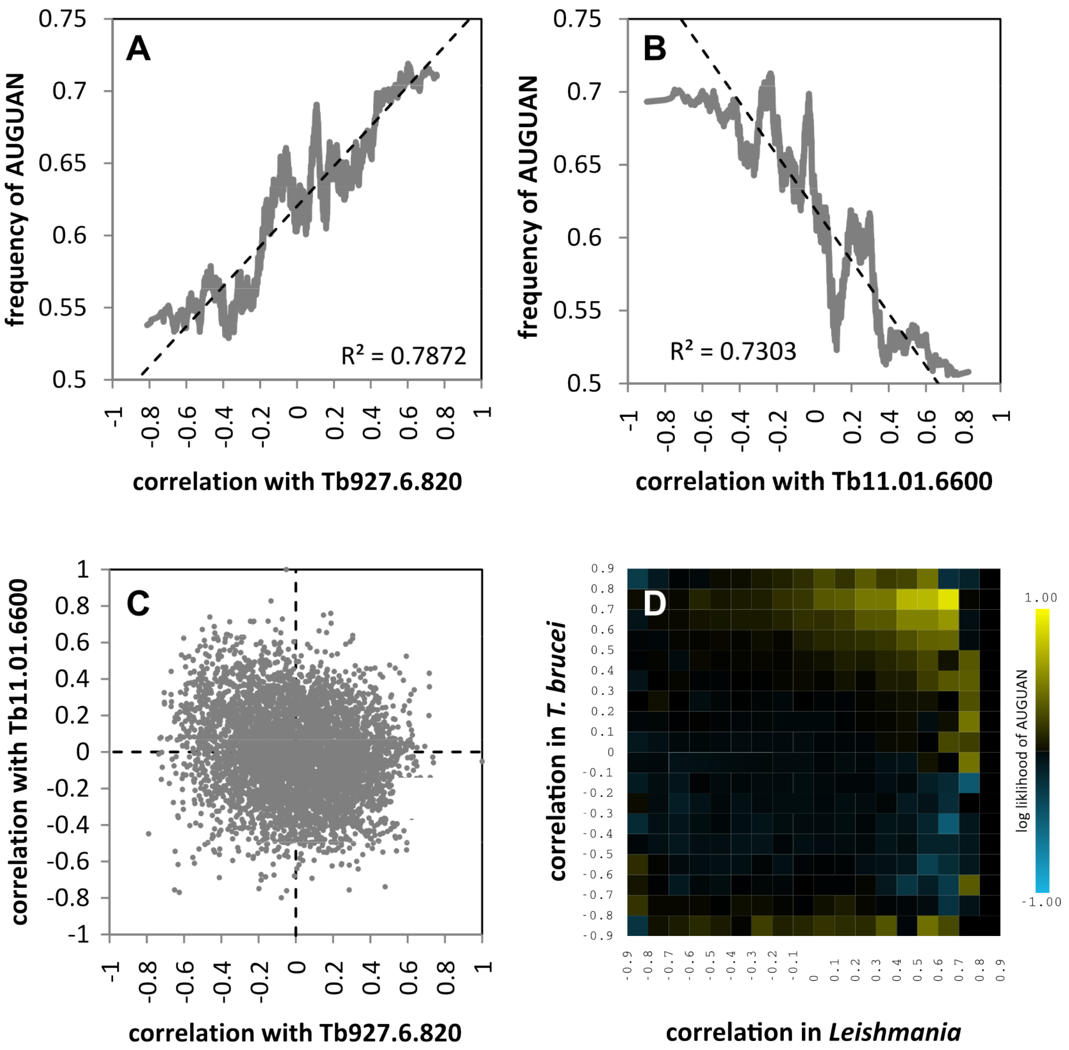

Supplementary Figure 5 – AUGUA as the potential binding sequence of PUF4 and PUF7. If a particular RNA-binding protein stabilizes (or destabilizes) the transcripts that harbor its binding sites, such transcripts should be correlated (or anti-correlated) with that RNA-binding protein (18), providing clues for the functional role of the RBPs in regulating abundance of their targets. Accordingly, meta-analysis of publicly available expression datasets (3-9) suggests stabilizing/destabilizing roles for two trypanosomatid proteins of the PUF family. The frequency of AUGUAN (CoRM17) is (A) significantly higher in transcripts that are positively correlated with PUF4 (Tb927.6.820, Mann-Whitney U z-score=7.12) and (B) in transcripts that are negatively correlated with PUF7 (Tb11.01.6600, Mann-Whitney U z-score=-8.46) across previously published expression datasets (3-9). However, (C) the binding sites of PUF4 and PUF7 potentially contain additional nucleotides that confer specificity, as transcripts that are positively correlated with PUF4 are not necessarily the same as transcripts that are negatively correlated with PUF7. (D) AUGUAN has conserved its regulatory role across trypanosomatids including both the *Trypanosoma* genus and *Leishmania* genus, as genes that are highly correlated in both *T. brucei* and *Leishmania spp.* are more likely to have conserved their regulatory neighborhood. Two genes are assumed to have conserved their regulatory neighborhood if both of them contain the AUGUAN motif in *T. brucei* as well as in all *Leishmania* species. To calculate the likelihood values, the set of such gene pairs were compared to a background set of gene pairs that are regulatory neighbors (i.e. both contain AUGUAN) in one organism, but have a broken regulatory neighborhood in other organisms (i.e. only one of them contains AUGUAN).

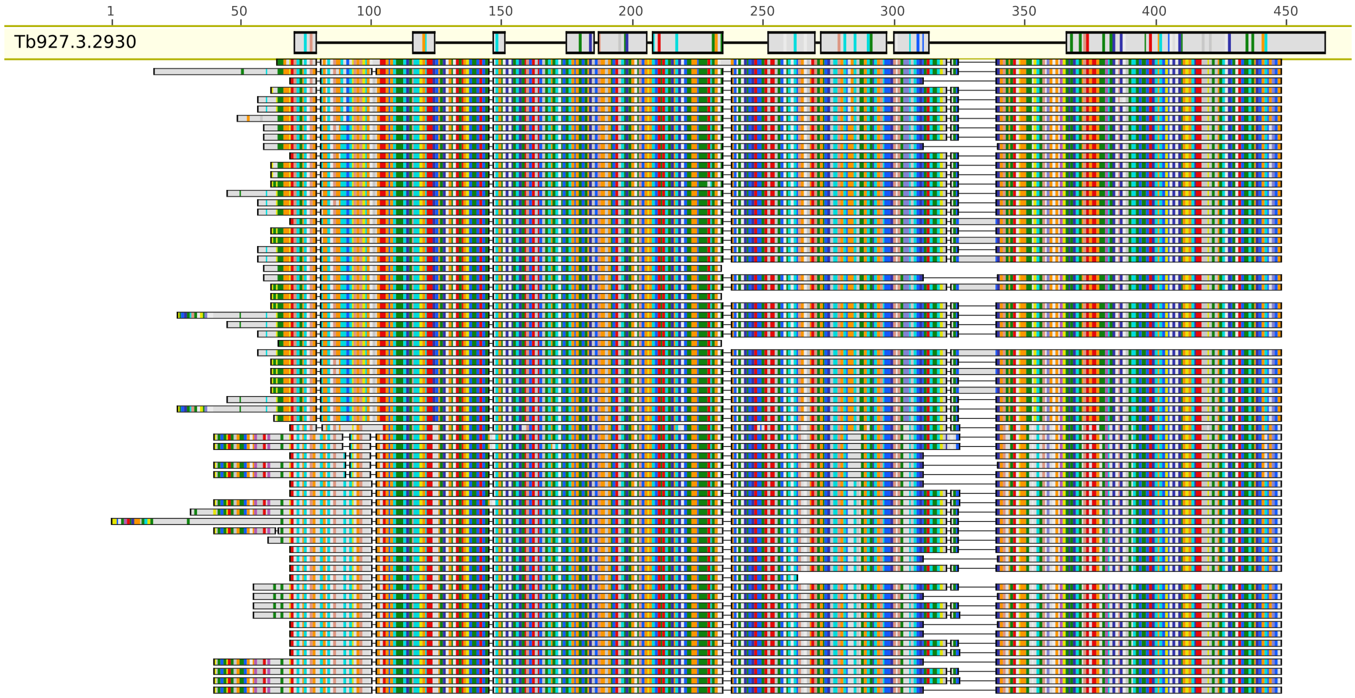

Supplementary Figure 6 – Alignment of *Tb*RBP6 (Tb927.3.2930) with ELAV-like proteins from non-trypanosomatids. The top hits from PSI-BLAST are shown below *Tb*RBP6, with conserved regions highlighted using RasMol color scheme (http://www.umass.edu/microbio/rasmol/distrib/rasman.htm#aminocolours). While the ELAV-like proteins that are found using PSI-BLAST are highly conserved, *Tb*RBP6 shows modest conservation (25.2% identity with consensus).

	Sequence	Genomic location (TriTrypDB v4)
**ARE**	AUUCUUUUCGAGUCUCUUUUAUUUAUUUAUUUAUUUAUUUUUUCUCUCUUAUU	Tb927_03_v4:1,112,163-1,112,215
**mutARE**	AUUCUUUUCGAGUCUCUUUUAUCUAUCUAUCUAUCUAUUUUUUCUCUCUUAUU	

Supplementary Figure 7 – RNA species that were used in gel-shift assays for determining sequence specificity of *Tb*RBP6. The ARE sequence was chosen from 566bp downstream of the stop codon of Tb927.3.3960, located on *T. brucei* chromosome 3. The four overlapping instances of the conserved motif CoRM1970 (AUUUAUU) are highlighted. In the mutant ARE (mutARE), one U of each CoRM1970 instance is mutated to C (underlined).

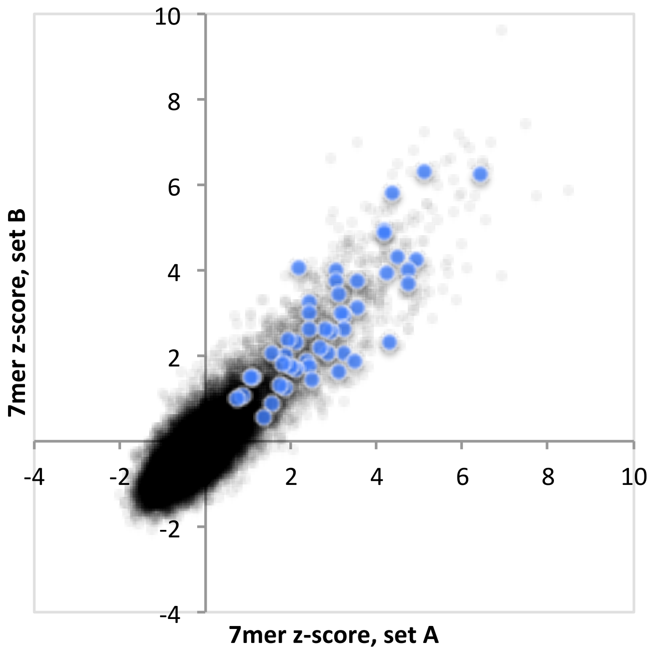

Supplementary Figure 8 – RNAcompete results for *T. brucei gambiense* protein Tbg972.7.6230, the homolog of Tb927.7.5380. Data are from ref. (18). Each axis represents the z-score of a distinct 7mer in one of the two independent probe sets used to identify RNA molecules that bind to Tbg972.7.6230 (16382 distinct 7mers, see ref. (19) for more details on RNAcompete assay and analysis). The blue dots represent the 7mers that contain AUUUA, the core recognition sequence of ELAV-like proteins. The larger z-scores of AUUUA-containing 7mers in both probe sets, compared to the rest of 7mers, suggest that Tbg972.7.6230 specifically recognizes AUUUA-containing sequences (Mann-Whitney U test p-value $< 2\times10^{-29}$).

Supplementary Tables

Supplementary Table 1 – All of the 388 non-redundant motifs identified in this study based on network-level conservation in the genus of *Trypanosoma*. In the third column, motif structure is denoted by dots and parenthesis, where dots represent unpaired nucleotides and matching parentheses represent paired nucleotides. For more details on motifs that were supported by microarray data, see Supplementary Table 2.

Motif	Sequence	Structure	Conservation *p*-value (sequence and structure)	Conservation *p*-value (sequence only)	Structure-dependent
CoRM1	CAUAGAN	....	2.12E-29	2.12E-29	NO
CoRM13	UNAUGGA	(.....)	1.43E-19	1.43E-19	NO
CoRM15	UYGANGA	((...))	1.64E-18	1.64E-18	NO
CoRM17	AUGUAN	......	3.65E-18	3.65E-18	NO
CoRM23	UYGCNGA	((...))	1.06E-17	1.06E-17	NO
CoRM25	UYCGNGA	((...))	3.95E-17	3.95E-17	NO
CoRM32	AUGGGNRU	((....))	2.03E-16	2.03E-16	NO
CoRM39	GCNCCNNNNGY	((.......))	5.02E-16	5.02E-16	NO
CoRM53	NNUCAGCUNNN	((.......))	4.05E-15	0.000291551	YES
CoRM60	UUYGNNNNCACGAA	((((......))))	1.83E-14	1.83E-14	NO
CoRM69	GANUGGNNNNNRNUY	((((.......))))	4.97E-14	1.59E-08	NO
CoRM80	NNCAGGANN	(.......)	9.42E-14	9.54E-07	NO
CoRM121	AUGUCNNNNNRYRU	((((......))))	8.60E-13	8.60E-13	NO
CoRM127	CNAAGGNNG	(.......)	1.24E-12	1.24E-12	NO
CoRM131	NNUYNACAAGANN	((((.....))))	1.42E-12	1.29E-06	NO
CoRM176	UGGCCNNNYYR	(((.....)))	4.81E-12	4.81E-12	NO
CoRM185	NRYUNNNCAAGUN	((((.....))))	6.86E-12	1.63E-07	NO
CoRM186	NYNCGCAAGN	((......))	6.99E-12	1.81E-08	NO
CoRM198	NGNUUNNNNNANAANCN	(((((.......)))))	1.09E-11	2.08E-05	NO
CoRM212	GGUNCANRYY	(((....)))	1.38E-11	1.38E-11	NO
CoRM234	NAGGCNAUN	((.....))	2.12E-11	6.33E-09	NO
CoRM264	NNUNNAGAACANN	(((.......)))	3.54E-11	9.23E-07	NO
CoRM298	NYAAGAAGN	((.....))	5.59E-11	1.12E-10	NO
CoRM306	NNUYGUNNACACGANN	((((((....))))))	6.21E-11	1.86E-08	NO
CoRM307	GCUCAGNNNNNNYUGRGY	(((((((....)))))))	6.21E-11	2.48E-10	NO
CoRM332	NNGRYNNNNNGUGUCNN	(((((.......)))))	8.46E-11	0.000107675	YES
CoRM360	GGUNNANUACC	(((.....)))	1.07E-10	1.07E-10	NO
CoRM384	NNNNRGRNNAAGUCUNNNN	(((((((.....)))))))	1.24E-10	8.35E-06	NO
CoRM429	GNCUGGAC	(......)	2.07E-10	2.07E-10	NO
CoRM444	NNGGGNNNNAAACCCNN	(((((.......)))))	2.48E-10	1.39E-07	NO
CoRM445	NNGUAUCCNRURYNN	((((((...))))))	2.48E-10	3.13E-07	NO

CoRM446	GYGRGUNNNACUCGC	((((((...))))))	2.48E-10	2.48E-10	NO
CoRM491	NNNNCAAGGANUGNNNN	((((((.....))))))	3.11E-10	0.000201617	YES
CoRM495	NAGNUNCNNNNNRNYUN	(((((.......)))))	3.20E-10	0.00403603	YES
CoRM505	GGYNNNNGAUGCC	(((.......)))	3.46E-10	3.46E-10	NO
CoRM509	NNNRNNAUNCUNNN	((((......))))	3.53E-10	0.00254345	YES
CoRM519	NUAUGAGNNNNYUYRURN	(((((((....)))))))	3.73E-10	2.98E-09	NO
CoRM541	NNYNNUACGAGNN	(((.......)))	4.37E-10	0.00119644	YES
CoRM580	ACUGGARGU	(((...)))	5.46E-10	5.46E-10	NO
CoRM591	NRUNNNNCGCAUN	(((.......)))	6.04E-10	2.72E-10	NO
CoRM594	GUGRNNUUCAC	((((...))))	6.08E-10	6.08E-10	NO
CoRM636	GGNCAGCC	((....))	7.87E-10	7.87E-10	NO
CoRM666	NCGGNUCNNNYYGN	((((......))))	9.85E-10	1.58E-06	NO
CoRM670	NGGAGCUNNNNNGYUYYN	((((((......))))))	9.94E-10	6.21E-08	NO
CoRM728	YUNAANCAG	((.....))	1.23E-09	1.23E-09	NO
CoRM765	YGYUYUNNNNAGAGCG	((((((....))))))	1.49E-09	1.49E-09	NO
CoRM776	NNNNNGRNCCNCUCNNNNN	(((((((.....)))))))	1.49E-09	5.47E-06	NO
CoRM783	YNUACCAG	(......)	1.62E-09	1.62E-09	NO
CoRM831	ANNUNGNNNNNNNYNRNNU	((((((.......))))))	1.98E-09	1.37E-07	NO
CoRM836	NNNNYGRNNCNCUCGNNNN	(((((((.....)))))))	1.99E-09	0.00409599	YES
CoRM866	GNUYUUNNNNAAGANC	((((((....))))))	2.24E-09	4.17E-08	NO
CoRM906	CCGUCGNNGRYGG	(((((...)))))	2.48E-09	2.48E-09	NO
CoRM918	RGYRGNNNCUGCU	(((((...)))))	2.60E-09	2.60E-09	NO
CoRM926	NNAUNAACNNNRUNN	((((.......))))	2.68E-09	6.93E-07	NO
CoRM947	NNCCGCAUYGGNN	(((((...)))))	2.98E-09	1.21E-06	NO
CoRM986	GCGCCGNNNNYGGYGY	((((((....))))))	3.35E-09	3.35E-09	NO
CoRM998	RNYUNNNNCGAGNU	((((......))))	3.44E-09	2.13E-06	NO
CoRM1003	UGYYNNUGGCA	((((...))))	3.50E-09	3.50E-09	NO
CoRM1016	CCAGGCNUGG	(((....)))	3.73E-09	3.73E-09	NO
CoRM1018	NNNNGNNUCAAACNNNN	(((((.......)))))	3.73E-09	0.00424737	YES
CoRM1019	NGUACGGNNNNNYGURYN	((((((......))))))	3.73E-09	6.26E-08	NO
CoRM1032	NNNNNNACCUNGNNNNNNN	((((((.......))))))	3.98E-09	0.000266377	YES
CoRM1043	NNAGCANAUNN	(((.....)))	4.11E-09	0.00475076	YES
CoRM1081	NYYRRNNNNNAUUGGN	(((((......)))))	4.51E-09	1.36E-08	NO
CoRM1091	NNGAUGCNCNN	(((.....)))	4.70E-09	8.27E-10	NO
CoRM1139	NYYUNAAGGN	(((....)))	5.42E-09	2.45E-05	NO
CoRM1188	NCGGGCUNNNNNYYYGN	(((((.......)))))	5.96E-09	5.22E-08	NO
CoRM1192	UYNUGRNNNNNUCANGA	((((((.....))))))	5.96E-09	1.88E-07	NO
CoRM1293	CGANCUNNNNNNGNUYG	(((((.......)))))	7.45E-09	2.61E-07	NO
CoRM1311	NNNNGUGGAUNYRYNNNN	(((((((....)))))))	7.83E-09	2.91E-07	NO
CoRM1359	UYNGGNNNGCCNGA	(((((....)))))	8.94E-09	2.52E-07	NO
CoRM1363	NNNAUGGUGNNYRUNNN	((((((.....))))))	8.94E-09	0.000576733	YES

CoRM1369	NGRNYNGNNNNNNNCNGNUCN	(((((((.......)))))))	8.94E-09	0.000644473	YES
CoRM1374	NUNUUNNCNAANAN	(((((....)))))	9.19E-09	0.000425128	YES
CoRM1422	NNNNYGUNGAGNNNN	(((((.....)))))	1.02E-08	4.17E-06	NO
CoRM1423	NNGANGCNNUYNN	(((((...)))))	1.02E-08	3.46E-10	NO
CoRM1436	NNNNNNNGGCGANNNNNNNNN	(((((((.......)))))))	1.04E-08	0.0287297	YES
CoRM1447	NYGNNNUNAACGN	(((.......)))	1.07E-08	0.000887285	YES
CoRM1450	YYRRNNUUUGG	((((...))))	1.07E-08	1.07E-08	NO
CoRM1455	NNYGNNAAGNCGNN	((((......))))	1.10E-08	0.00144562	YES
CoRM1515	NNNYYGRNNNNCUCGGNNN	(((((((.....)))))))	1.19E-08	5.83E-05	YES
CoRM1534	UGYYNNNNGGGCA	((((.....))))	1.25E-08	1.25E-08	NO
CoRM1615	NGCCGUANNGGYN	((((.....))))	1.49E-08	1.05E-06	NO
CoRM1617	CAUGGGNNNNYYYRUG	((((((....))))))	1.49E-08	1.49E-08	NO
CoRM1618	CCNAUGNNNNNYRUNGG	((((((.....))))))	1.49E-08	1.41E-14	NO
CoRM1663	NNUGGCACNNNNNGYYRNN	((((((.......))))))	1.68E-08	1.77E-05	NO
CoRM1664	NNYGGNNNNNNAANCCGNN	((((((.......))))))	1.68E-08	0.000171728	YES
CoRM1691	CCAUACNNNRUGG	((((.....))))	1.79E-08	1.79E-08	NO
CoRM1694	NNNNGGNNNCANACCNNNN	((((((.......))))))	1.79E-08	0.00366707	YES
CoRM1725	NNNNNGNUGGUGCNNNNN	((((((......))))))	1.86E-08	0.0104629	YES
CoRM1727	URYGNGNNNNNNNCNCGUA	((((((.......))))))	1.86E-08	4.93E-07	NO
CoRM1773	NYUURRNNNNNNNNUUAAGN	((((((.......))))))	2.01E-08	1.97E-06	NO
CoRM1810	NNRUUCNGAAUNN	(((((...)))))	2.17E-08	8.21E-05	YES
CoRM1829	NCCGUUCNNNNYGGN	((((.......))))	2.24E-08	1.20E-06	NO
CoRM1837	NNNNNGCUUCANNYNNNNN	((((((.......))))))	2.24E-08	0.175109	YES
CoRM1882	NNGUNAAGAACNN	((((.....))))	2.48E-08	1.40E-09	NO
CoRM1909	NNNNAGCNGGNGYUNNNN	(((((((....)))))))	2.61E-08	4.54E-06	NO
CoRM1910	NNNNANCUNGNGNUNNNN	(((((((....)))))))	2.61E-08	2.24E-05	NO
CoRM1934	NNNNGANGGCNNNNNUYNNNN	(((((((.......)))))))	2.68E-08	0.00657004	YES
CoRM1950	GCNCAGNNNNNNYUGNGY	((((((......))))))	2.78E-08	3.49E-07	NO
CoRM1953	GCNNAGNNNNNNYUNNGY	(((((((....)))))))	2.80E-08	0.00845982	YES
CoRM1970	AUUUAUU	(.....)	2.86E-08	2.86E-08	NO
CoRM2002	NNNCGUAAGGNNN	((((.....))))	2.98E-08	0.00226332	YES
CoRM2004	NGUAUCCNNNRURYN	(((((.....)))))	2.98E-08	3.49E-07	NO
CoRM2009	NCNCAGNNNNNNYUGNGN	(((((((....)))))))	2.98E-08	0.0012397	YES
CoRM2010	NNYYRGYNNNNGCUGGNN	(((((((....)))))))	2.98E-08	2.11E-05	NO
CoRM2239	NRGYNCGGGCUN	((((....))))	4.17E-08	1.78E-06	NO
CoRM2240	NGAUAGGNNNRUYN	((((......))))	4.17E-08	1.14E-06	NO
CoRM2279	AGUCGUNGRYU	((((...))))	4.47E-08	4.47E-08	NO
CoRM2281	NNYGUNCACACGNN	(((((....)))))	4.47E-08	2.99E-05	NO
CoRM2283	NGRYGNNNNGCCGUCN	(((((......)))))	4.47E-08	1.05E-06	NO
CoRM2284	UGGAACNNNNNNUUYYR	(((((.......)))))	4.47E-08	4.47E-08	NO
CoRM2335	GNGRGNNNNACUCNC	(((((.....)))))	4.77E-08	2.85E-06	NO

CoRM2405	NNUNAANCCANN	(((......)))	5.29E-08	2.05E-05	NO
CoRM2447	NNGANCCAYNN	(((.....)))	5.58E-08	0.194717	YES
CoRM2454	GCNAGUNNNRYUNGY	((((((...))))))	5.59E-08	1.31E-06	NO
CoRM2469	NUCAGNUNNNNYUGRN	((((((....))))))	5.74E-08	8.01E-05	YES
CoRM2534	YGGYNNGCGCCG	((((....))))	6.21E-08	6.21E-08	NO
CoRM2553	UYGNNUACGA	(((....)))	6.33E-08	6.33E-08	NO
CoRM2597	YGRGUNNNACUCG	(((((...)))))	6.71E-08	6.71E-08	NO
CoRM2600	NNNGGCUGANNYYNNN	(((((......)))))	6.71E-08	0.00566673	YES
CoRM2604	UGCGGGNNNNNNNYYYGYR	((((((.......))))))	6.71E-08	6.71E-08	NO
CoRM2698	URNNGGNNNNNNNCCNNUA	((((((.......))))))	7.61E-08	0.000169706	YES
CoRM2720	GYGGNAACCGC	((((...))))	7.83E-08	7.83E-08	NO
CoRM2751	GUUUGANNNNNNNUYRRRY	((((((.......))))))	8.05E-08	8.05E-08	NO
CoRM2843	GYYNGNNNACNGGC	(((((....)))))	8.94E-08	7.21E-06	NO
CoRM2863	GUANUCNNNNGRNURY	((((((....))))))	9.13E-08	7.38E-07	NO
CoRM2871	CAAAAC	......	9.16E-08	9.16E-08	NO
CoRM2933	NNGGNUACNYYNN	((((.....))))	1.01E-07	0.000316367	YES
CoRM2967	ACAUGGNNNNNRUGU	((((.......))))	1.04E-07	1.04E-07	NO
CoRM2968	NNNUNNGGGCAANNN	((((.......))))	1.04E-07	0.0102392	YES
CoRM3033	GACUACNUY	((.....))	1.14E-07	1.14E-07	NO
CoRM3049	GYYGNNNNNUUCGGC	((((.......))))	1.15E-07	1.15E-07	NO
CoRM3050	NNNNUGANGUNNUYRNNNNN	(((((((.....)))))))	1.15E-07	2.47E-05	NO
CoRM3064	NNUCNGCUNNNNYNGRNN	((((((......))))))	1.17E-07	0.00544856	YES
CoRM3065	YYRRUNNNNNNNNNNAUUGG	(((((((.....)))))))	1.17E-07	6.69E-05	YES
CoRM3078	CGAUGUNNNNNYRUYG	(((((......)))))	1.19E-07	1.19E-07	NO
CoRM3079	NNRUYNNNNAANGAUNN	((((((.....))))))	1.19E-07	2.12E-05	NO
CoRM3102	NNACNAACNNGUNN	(((((....)))))	1.22E-07	0.00835425	YES
CoRM3108	NNNNCGGNAUNNYGNNNN	((((((......))))))	1.22E-07	0.00251532	YES
CoRM3146	NGNUACARNYN	((((...))))	1.26E-07	2.00E-06	NO
CoRM3150	NNAUAGUUNNNRUNN	((((.......))))	1.27E-07	0.000659621	YES
CoRM3163	NNNAGCUGNNGYUNNN	((((((....))))))	1.28E-07	2.30E-05	NO
CoRM3209	GRRYRNNNNNAUGUUC	(((((......)))))	1.34E-07	1.34E-07	NO
CoRM3227	UGRNUNAUCA	(((....)))	1.36E-07	1.36E-07	NO
CoRM3296	CGUACUNNNNNURYG	((((......))))	1.43E-07	1.43E-07	NO
CoRM3359	UGGAUGNNNNNYRUYYR	((((((.....))))))	1.52E-07	1.52E-07	NO
CoRM3368	GNRUYNNNNAGAUNC	(((((.....)))))	1.53E-07	7.54E-06	NO
CoRM3378	NUGUAUCNNNURYRN	(((((.....)))))	1.55E-07	4.07E-06	NO
CoRM3391	NNCAUCGUNUGNN	((((.....))))	1.57E-07	0.000174799	YES
CoRM3393	NNYGUCNAACGNN	(((((...)))))	1.57E-07	0.000232195	YES
CoRM3489	NAUGANANNNNNNUYRUN	((((((......))))))	1.70E-07	0.000619992	YES
CoRM3558	NGUUUUGNRRYN	((((....))))	1.82E-07	2.29E-06	NO
CoRM3596	NNURNNCCCUUANN	((((......))))	1.86E-07	0.000238906	YES

CoRM3708	AUCAAGNNNNUGRU	((((......))))	2.09E-07	2.09E-07	NO
CoRM3709	YRUYGNNNNNGCGAUG	(((((......)))))	2.09E-07	2.09E-07	NO
CoRM3762	NNAUUCAGNNN	((.......))	2.17E-07	0.000242812	YES
CoRM3787	UGGYNNNNNGNGCCA	(((((.....)))))	2.23E-07	8.28E-06	NO
CoRM3830	UGNCNUACA	((.....))	2.31E-07	2.31E-07	NO
CoRM3880	NNNACUCCCNUNNN	((((......))))	2.39E-07	0.0247597	YES
CoRM3907	YUUYRNNAUGAAG	(((((...)))))	2.46E-07	2.46E-07	NO
CoRM3921	NNGGNNUANACCNN	((((......))))	2.48E-07	0.000635673	YES
CoRM3926	NNNNUNNAACUNANNNN	(((((.......)))))	2.50E-07	0.056462	YES
CoRM3981	CGNAUCNNNNNYG	(((.......)))	2.61E-07	8.76E-06	NO
CoRM4080	NNYGUNNNNCCNACGNN	(((((.......)))))	2.78E-07	0.000447124	YES
CoRM4119	CCGGUGNNNYYGG	((((.....))))	2.86E-07	2.86E-07	NO
CoRM4197	UGYCNAGCA	(((...)))	3.03E-07	3.03E-07	NO
CoRM4227	CGCGGUNYG	((.....))	3.11E-07	3.11E-07	NO
CoRM4238	RUGGYNNNNNGCCAU	(((((.....)))))	3.13E-07	3.13E-07	NO
CoRM4323	GCANGGNNNYYNUGY	((((((...))))))	3.34E-07	9.20E-06	NO
CoRM4393	NNCUACCCNGNN	(((......)))	3.52E-07	0.000401341	YES
CoRM4477	NGUNNNAGUUACN	(((.......)))	3.76E-07	1.78E-05	NO
CoRM4479	UACGACNNYGUR	((((....))))	3.76E-07	3.76E-07	NO
CoRM4485	NNNAGGACAUNNN	((((.....))))	3.76E-07	5.19E-05	YES
CoRM4513	GAUUCUNNNRRUY	((((.....))))	3.88E-07	3.88E-07	NO
CoRM4517	UGACNGA	(.....)	3.88E-07	3.88E-07	NO
CoRM4559	RRRNNNGAGUUU	(((......)))	3.99E-07	3.99E-07	NO
CoRM4625	NNGYNANGNGCNN	(((((...)))))	4.20E-07	1.59E-06	NO
CoRM4628	CCCGUAGG	((....))	4.21E-07	4.21E-07	NO
CoRM4688	NYUGNNNUCGCAGN	((((......))))	4.35E-07	9.12E-06	NO
CoRM4715	NNNUNUGGAANANNN	((((((...))))))	4.43E-07	7.44E-05	YES
CoRM4769	RUYUNNNNGGAGAU	((((......))))	4.59E-07	4.59E-07	NO
CoRM4834	NGCGGNCNNNNYYGYN	((((((....))))))	4.78E-07	0.000323415	YES
CoRM4889	NNAUACAANRUNN	((((.....))))	4.92E-07	0.00139358	YES
CoRM4905	YUGYRNNCUGCAG	(((((...)))))	4.97E-07	4.97E-07	NO
CoRM4909	NYUUNCACAAGN	((((....))))	5.01E-07	4.09E-05	NO
CoRM4919	NNGGYNNGGNGCCNN	(((((.....)))))	5.03E-07	0.000881915	YES
CoRM4923	NUUUGGCNNNNYYRRRN	((((((.....))))))	5.03E-07	7.97E-06	NO
CoRM4928	NNNNNGCGGGCNNNN	((((.......))))	5.07E-07	0.111492	YES
CoRM4983	NNNNGCUNGCNNGYNNNN	((((((......))))))	5.22E-07	0.000349604	YES
CoRM5069	CAGGGNNNYUG	(((.....)))	5.53E-07	5.53E-07	NO
CoRM5078	NNNNNNGNCCNCYNNNNNN	(((((((.....)))))))	5.57E-07	0.0226062	YES
CoRM5103	NNNNGGCGANNNYYNNNN	((((((......))))))	5.64E-07	0.345663	YES
CoRM5171	RUYYGNNNCGGAU	(((((...)))))	5.84E-07	5.84E-07	NO
CoRM5227	NNNNGNNCCGNGCNNNN	(((((.......)))))	6.01E-07	0.0282279	YES

CoRM5228	UCCAGANNNNGGR	(((.......)))	6.03E-07	6.03E-07	NO
CoRM5254	GYYYNNNCUGGGC	((((.....))))	6.10E-07	6.10E-07	NO
CoRM5292	CUUUGGNNYRRRG	(((((...)))))	6.20E-07	6.20E-07	NO
CoRM5409	NGUNNNAAGCACN	(((.......)))	6.64E-07	4.50E-05	NO
CoRM5416	NGCANGGNNNYNUGYN	((((((....))))))	6.68E-07	0.00033097	YES
CoRM5562	GYGNGNNNNNCNCGC	((((((...))))))	7.24E-07	0.00113121	YES
CoRM5649	NNNNNNAUGAUGNNNNN	(((((.......)))))	7.51E-07	1.51E-08	NO
CoRM5728	NGCGCNNNNNNNGYGYN	(((((((...)))))))	7.83E-07	0.0217762	YES
CoRM5805	NUYUGCAAGAN	((((...))))	8.14E-07	1.82E-05	NO
CoRM5827	NNYUYUNNUNAGAGNN	((((((....))))))	8.29E-07	0.000224327	YES
CoRM5914	NAUGCGNNNNNYRUN	((((.......))))	8.71E-07	0.000712759	YES
CoRM5939	NNNNNGUUACANNNN	((((.......))))	8.79E-07	0.113111	YES
CoRM5987	NGRRNUACUUCN	((((....))))	9.02E-07	4.28E-05	NO
CoRM6030	GRGNCACCUC	(((....)))	9.22E-07	9.22E-07	NO
CoRM6126	NGGUNNCNCACCN	((((.....))))	9.69E-07	5.03E-05	YES
CoRM6152	NCGUNGURYGN	((((...))))	9.82E-07	2.96E-05	NO
CoRM6200	GYYRNNNNNAUGGC	((((......))))	1.01E-06	1.01E-06	NO
CoRM6217	UNGYRYNNNNGUGCNA	((((((....))))))	1.02E-06	2.23E-05	NO
CoRM6261	NYUUYNNNNNAUGAAGN	(((((.......)))))	1.04E-06	1.70E-05	NO
CoRM6302	NNGUCNGNNNNNYNGRYNN	(((((((.....)))))))	1.07E-06	0.0174916	YES
CoRM6345	NCAUCAUNNNRUGN	((((......))))	1.09E-06	2.64E-05	NO
CoRM6362	GGYRNGAUGCC	((((...))))	1.10E-06	1.10E-06	NO
CoRM6465	NRGRNNNNNCANUCUN	(((((......)))))	1.14E-06	0.00259682	YES
CoRM6488	YRUYRYNNNNNNNGUGAUG	((((((.......))))))	1.16E-06	1.16E-06	NO
CoRM6602	GCAUGGNRUGY	((((...))))	1.22E-06	1.22E-06	NO
CoRM6650	NNNGAAUUGNYNNN	((((......))))	1.25E-06	0.220239	YES
CoRM6693	NNNNAGACNANNN	(((.......)))	1.27E-06	6.64E-05	YES
CoRM6763	NYRUYNNNUGGAUGN	(((((.....)))))	1.31E-06	4.68E-05	NO
CoRM6764	NAGNNCANNNNNNGNNYUN	((((((.......))))))	1.31E-06	0.0846544	YES
CoRM6864	CAUGCANNNNNGYRUG	(((((......)))))	1.38E-06	1.38E-06	NO
CoRM6870	NGUNNAANNACN	((((....))))	1.38E-06	0.0121621	YES
CoRM6871	NGACCGCUYN	(((....)))	1.38E-06	0.000143047	YES
CoRM6887	NNGGACCACNN	(((.....)))	1.39E-06	0.00276236	YES
CoRM7029	GUGGGGNNNYYYYRY	((((((...))))))	1.47E-06	1.47E-06	NO
CoRM7127	AGUCNGNNNNRYU	(((.......)))	1.53E-06	1.53E-06	NO
CoRM7129	YRUGNNNGACAUG	((((.....))))	1.53E-06	1.53E-06	NO
CoRM7142	NNGYUNNGGNAGCNN	(((((.....)))))	1.54E-06	0.00293888	YES
CoRM7200	GNCUCNAC	(......)	1.57E-06	1.57E-06	NO
CoRM7359	NNUNCCGUAANN	(((......)))	1.70E-06	0.00197858	YES
CoRM7443	NNGGNNNGNGACCNN	((((.......))))	1.75E-06	4.54E-06	NO
CoRM7492	GUGNNNNCAGCAC	(((.......)))	1.80E-06	1.80E-06	NO

CoRM7495	NNYGNNUACACGNN	((((......))))	1.80E-06	0.00230365	YES
CoRM7521	NGUNCGCCACN	(((.....)))	1.82E-06	9.80E-05	YES
CoRM7549	NNNGNNNUGGGNCNNN	(((((......)))))	1.84E-06	0.000137082	YES
CoRM7552	GUUYNNNNAGAAC	((((.....))))	1.85E-06	1.85E-06	NO
CoRM7649	UAGGCGNNYUR	(((.....)))	1.92E-06	1.92E-06	NO
CoRM7721	NNNNCAGUUNGNNNN	(((((.....)))))	1.97E-06	0.000326218	YES
CoRM7809	NNNNYRNGANCUGNNNN	((((((.....))))))	2.03E-06	2.77E-05	NO
CoRM7810	ANUGGUNNNNNNRYYRNU	(((((((....)))))))	2.03E-06	1.15E-07	NO
CoRM7840	NRGURNNUNUACUN	(((((....)))))	2.06E-06	6.20E-05	YES
CoRM7859	NNNNUGACANYRNNNN	((((((....))))))	2.08E-06	0.0126455	YES
CoRM7914	RGRYNNNNNNNANGUCU	(((((.......)))))	2.12E-06	6.69E-05	YES
CoRM7943	GGRNNNNCUCUCC	(((.......)))	2.15E-06	2.15E-06	NO
CoRM7977	NCCUNGGNNNRGGN	((((......))))	2.17E-06	0.000105933	YES
CoRM8019	NNGAUGCGNNUYNN	((((......))))	2.21E-06	0.00283139	YES
CoRM8124	YGGNGGGCCG	(((....)))	2.30E-06	2.30E-06	NO
CoRM8170	CUNAUUNNNNNNRUNRG	(((((.......)))))	2.35E-06	3.87E-05	NO
CoRM8225	GRGNRRNNNUUNCUC	((((((...))))))	2.41E-06	4.08E-05	NO
CoRM8241	CANCCANNNGNUG	((((.....))))	2.42E-06	0.000188702	YES
CoRM8259	GRNUGCAUC	((.....))	2.43E-06	2.43E-06	NO
CoRM8509	RRGYNNNNCAGCUU	((((......))))	2.66E-06	2.66E-06	NO
CoRM8525	NGCAGCANUGYN	((((....))))	2.67E-06	0.000162666	YES
CoRM8680	NNGGCNCCNNGYYNN	(((((.....)))))	2.82E-06	0.0039588	YES
CoRM8756	NYRUNRNNUUNAUGN	((((((...))))))	2.89E-06	0.00778467	YES
CoRM8941	CGAACNNNNUUYG	((((.....))))	3.07E-06	3.07E-06	NO
CoRM8962	GUUAUUNNRRY	(((.....)))	3.10E-06	3.10E-06	NO
CoRM9227	NAGNCUANNNNNYUN	((((.......))))	3.38E-06	1.04E-05	NO
CoRM9289	NNACNGGUNNNGUNN	((((.......))))	3.45E-06	0.00815134	YES
CoRM9336	NNANUUNGNNNNNRRNUNN	(((((((.....)))))))	3.51E-06	0.000608797	YES
CoRM9505	GGNNCCGCCC	((......))	3.71E-06	3.71E-06	NO
CoRM9518	RYYUYNNNNNNGAGGU	((((((....))))))	3.73E-06	0.000212367	YES
CoRM9559	NNCUGGGUGNN	(((.....)))	3.78E-06	0.0131605	YES
CoRM9654	YUUNGGCAAG	(((....)))	3.90E-06	3.90E-06	NO
CoRM9676	YUGAGCAG	((....))	3.93E-06	3.93E-06	NO
CoRM9692	UUCGCCNGRR	(((....)))	3.95E-06	3.95E-06	NO
CoRM9710	NCGGACCNGN	((......))	3.97E-06	0.000174403	YES
CoRM9751	NGGUCGCNNNRYYN	((((......))))	4.01E-06	0.000143025	YES
CoRM9755	ANCGNGNNYGNU	((((....))))	4.01E-06	0.00015403	YES
CoRM9862	NGAGGACYUYN	((((...))))	4.18E-06	0.000154959	YES
CoRM9912	NNRNGGNNUCCNUNN	((((((...))))))	4.28E-06	0.194017	YES
CoRM9966	NNGGAUAANNYYNN	((((......))))	4.36E-06	0.0037179	YES
CoRM10028	RGUGNNNNCCACU	((((.....))))	4.46E-06	4.46E-06	NO

CoRM10064	AUNCGNNNNYGNRU	(((((....)))))	4.51E-06	0.00132031	YES
CoRM10308	NGGACAUNNNNUYYN	((((.......))))	4.83E-06	0.000113459	YES
CoRM10419	RUYNUCCGAU	(((....)))	5.03E-06	5.03E-06	NO
CoRM10430	NCAAUGANUGN	(((.....)))	5.05E-06	0.000183325	YES
CoRM10436	GYGAGACGC	(((...)))	5.06E-06	5.06E-06	NO
CoRM10471	GNUGCANNNNNUGYRNY	((((((.....))))))	5.10E-06	1.59E-08	NO
CoRM10472	UCGGCCNNNNYGR	(((.......)))	5.10E-06	5.10E-06	NO
CoRM10555	CGNCCGNGNYG	((((...))))	5.26E-06	0.00030061	YES
CoRM10574	YGGCCACCG	(((...)))	5.30E-06	5.30E-06	NO
CoRM10592	NNUNUGGUCANN	(((......)))	5.32E-06	0.012096	YES
CoRM10871	NNNNGRRNNNNANNUUCNNNN	(((((((.......)))))))	5.72E-06	0.0547071	YES
CoRM10877	GUYNNNNGUGGAC	(((.......)))	5.74E-06	5.74E-06	NO
CoRM10978	YUNGNNGGCNAG	((((....))))	5.90E-06	0.000159964	YES
CoRM11049	NCCGNCUNNYGGN	((((.....))))	6.02E-06	0.000263417	YES
CoRM11124	GYYRYNNNGGUGGC	(((((....)))))	6.12E-06	6.12E-06	NO
CoRM11132	NYGNNNUCCCCGN	(((.......)))	6.14E-06	0.000486648	YES
CoRM11205	GGAUNNNNNNNNNNNNNRUYY	(((((((.......)))))))	6.26E-06	0.000331528	YES
CoRM11529	CUGGCUNYRG	(((....)))	6.85E-06	6.85E-06	NO
CoRM11628	GRRUNNNCNAUUC	((((.....))))	7.04E-06	7.04E-06	NO
CoRM11636	NNNNNNNNNCCCGNNNNNN	((((((.......))))))	7.06E-06	0.00926905	YES
CoRM11698	GRYYNNGGGUC	((((...))))	7.20E-06	7.20E-06	NO
CoRM11997	NYYRNYNNNNNNAGNUGGN	((((((.......))))))	7.75E-06	0.00780217	YES
CoRM12076	NGYNNUNNNNNCANNGCN	((((((......))))))	7.92E-06	0.39183	YES
CoRM12102	NNNNGRNANNAUCNNNN	((((((.....))))))	7.97E-06	0.000565038	YES
CoRM12122	NUGYNNNGCAGCAN	((((......))))	8.02E-06	0.000394556	YES
CoRM12343	NNNGUCNGANRYNNN	(((((.....)))))	8.50E-06	4.11E-05	NO
CoRM12496	GUUNNNNNGNNAAC	(((((....)))))	8.85E-06	6.72E-05	YES
CoRM12558	NNNNGAGNNGNYUYNNNN	(((((((....)))))))	8.95E-06	0.00022996	YES
CoRM12631	YNGNNNUGCCNG	(((......)))	9.10E-06	3.45E-08	NO
CoRM12697	AGACGGNNU	(.......)	9.27E-06	9.27E-06	NO
CoRM12831	NGCUNUUNNNRNRGYN	((((((....))))))	9.52E-06	0.00848764	YES
CoRM12841	GYUYNNNNAGGAGC	((((......))))	9.56E-06	9.56E-06	NO
CoRM12948	UUGUUGNNNNNNNYRRYRR	(((((((.....)))))))	9.80E-06	0.000306094	YES
CoRM12969	GYRRGNNNCUUGC	(((((...)))))	9.84E-06	9.84E-06	NO
CoRM13050	NYNGAUACNGN	((((...))))	1.00E-05	0.000196313	YES
CoRM13101	CGUGCUNNNNNNYRYG	((((........))))	1.01E-05	1.01E-05	NO
CoRM13118	NNRYNAACAGUNN	((((.....))))	1.02E-05	0.0156895	YES
CoRM13506	YGUNNNNGUCACG	(((.......)))	1.12E-05	1.12E-05	NO
CoRM13522	AACNUANNNNGUU	(((.......)))	1.12E-05	1.12E-05	NO
CoRM13677	NNNGAACUNUYNNN	(((((....)))))	1.17E-05	0.188556	YES
CoRM13701	NNNNUCNAGURNNNN	(((((.....)))))	1.17E-05	0.438242	YES

CoRM13749	GACAACC	(.....)	1.19E-05	1.19E-05	NO
CoRM13832	NAACAUGUUN	(((....)))	1.21E-05	0.000352276	YES
CoRM14266	NGGUCAANYYN	(((.....)))	1.31E-05	0.00053539	YES
CoRM14286	NGGANUCNNUYYN	(((((...)))))	1.32E-05	0.0139244	YES
CoRM14482	NUYRNCAUUGAN	((((....))))	1.38E-05	0.000442055	YES
CoRM14520	GYNNAGAAGC	((......))	1.39E-05	1.39E-05	NO
CoRM14585	NCNAGNCUNGN	((((...))))	1.41E-05	0.000249175	YES
CoRM14648	NNNAUNAGGNNUNNN	((((.......))))	1.43E-05	0.0183699	YES
CoRM14683	NGCACUANNNGYN	(((.......)))	1.44E-05	0.000342053	YES
CoRM14703	NNGGUNNCNNNNNRYYNN	(((((((....)))))))	1.44E-05	0.0345405	YES
CoRM14755	NNURNNNGUCNUANN	((((.......))))	1.46E-05	0.0203366	YES
CoRM14907	CAUGACRUG	(((...)))	1.51E-05	1.51E-05	NO
CoRM14967	NYYNRYNNNNNCGUNGGN	((((((......))))))	1.52E-05	0.00704939	YES
CoRM15195	NGAUCCNRUYN	((((...))))	1.59E-05	0.000563353	YES
CoRM15269	NNYGUNNNNNCNNACGNN	((((((......))))))	1.61E-05	0.046329	YES
CoRM15402	NNGNNCAGCGCNN	(((.......)))	1.65E-05	0.022509	YES
CoRM15491	NNNGYRNNNNNNGGUGCNNN	((((((.......))))))	1.68E-05	0.034878	YES
CoRM15495	GYRRNCUUUGC	((((...))))	1.68E-05	1.68E-05	NO
CoRM15588	YGNUNGAANCG	((((...))))	1.70E-05	0.000693173	YES
CoRM15706	AAGGCNYUU	(((...)))	1.73E-05	1.73E-05	NO
CoRM15809	ACUUNANNNRRGU	((((.....))))	1.78E-05	1.78E-05	NO
CoRM15823	NURRYNANGUUAN	(((((...)))))	1.78E-05	0.000617086	YES
CoRM15869	GUCGCANNNNGRY	(((.......)))	1.79E-05	1.79E-05	NO
CoRM15987	NUGGACNNNNUYYRN	(((((.....)))))	1.83E-05	4.20E-07	NO
CoRM15998	NUNCCGNCAN	((......))	1.84E-05	0.00196215	YES
CoRM16101	NNACUUCANNUNN	(((.......)))	1.87E-05	0.0719592	YES
CoRM16121	NNNNGAUUACNNN	(((.......)))	1.87E-05	0.000141372	YES
CoRM16238	UANGACNNNNYNUR	((((......))))	1.91E-05	0.000839546	YES
CoRM16614	NNRRNNNUGGAUUNN	((((.......))))	2.06E-05	0.0189975	YES
CoRM16983	NNGUANCGNURYNN	(((((....)))))	2.19E-05	0.0159233	YES
CoRM17098	AGCAGNNUGYU	((((...))))	2.23E-05	2.23E-05	NO
CoRM17183	GYYNYYNNNNNNGGNGGC	((((((......))))))	2.27E-05	0.000650286	YES
CoRM17203	NNYRNNNCAACUGNN	((((.......))))	2.27E-05	0.0441853	YES
CoRM17349	NNNNNNUCUGNUNNNNN	(((((.......)))))	2.33E-05	0.516376	YES
CoRM17370	AUCUCGRU	((....))	2.33E-05	2.33E-05	NO
CoRM17476	AGUACANRYU	(((....)))	2.38E-05	2.38E-05	NO
CoRM17656	UGYACGGCA	(((...)))	2.45E-05	2.45E-05	NO
CoRM17670	NNNAANCNAUUNNN	(((((....)))))	2.46E-05	0.159815	YES
CoRM17727	NNNANAGCCNNUNNN	((((.......))))	2.48E-05	0.0304499	YES
CoRM17771	NCUGGCANNRGN	(((......)))	2.50E-05	0.00110732	YES
CoRM17941	NNUUNNNGCGNAANN	((((.......))))	2.58E-05	0.0302369	YES

CoRM17945	NNNYYNGGUNGGNNN	(((((.....)))))	2.58E-05	0.0639509	YES
CoRM17951	NNACANCAGUNN	((((....))))	2.58E-05	0.0522543	YES
CoRM18023	GGYNUANGCC	(((....)))	2.61E-05	2.61E-05	NO
CoRM18026	NCAGGCNNNNYUGN	((((......))))	2.61E-05	0.000754074	YES
CoRM18467	AGGCNGNNNGYYU	((((.....))))	2.80E-05	2.80E-05	NO
CoRM18494	GCCUNUNNNNNRGGY	((((.......))))	2.81E-05	2.81E-05	NO
CoRM18807	UNNAAGNNNNNNNYUUNNR	((((((.......))))))	2.95E-05	0.0291851	YES
CoRM18974	GAAUUANNNNUUY	(((.......)))	3.03E-05	3.03E-05	NO
CoRM19174	GUYRNNNNNAUGAC	((((......))))	3.13E-05	3.13E-05	NO
CoRM19396	NNNNNRYNNNNANGGUNNNNN	(((((((.......)))))))	3.23E-05	0.00321787	YES
CoRM19412	NAUGGCANRUN	(((.....)))	3.24E-05	0.00105029	YES
CoRM19445	GURNNNNCANUAC	((((.....))))	3.25E-05	0.000892911	YES
CoRM19544	NCNGAGCNNNNYNGN	((((.......))))	3.30E-05	0.0406554	YES
CoRM19642	NUYNNGCGGGAN	(((......)))	3.36E-05	0.00135465	YES
CoRM19982	NNYUNYNNNGNGNAGNN	(((((((...)))))))	3.51E-05	0.0480054	YES
CoRM19983	NUAGUUGNNURN	(((......)))	3.52E-05	0.00113791	YES
CoRM20057	URNCUACUA	((.....))	3.56E-05	3.56E-05	NO
CoRM20588	NUGGUUCYYRN	((((...))))	3.84E-05	5.93E-07	NO
CoRM20807	NUYNAGGUGAN	(((.....)))	3.94E-05	0.00150963	YES
CoRM20879	NNRNAAGUCUNN	(((......)))	3.98E-05	0.0332516	YES
CoRM20955	NNYGNAUCCGNN	((((....))))	4.03E-05	0.0395853	YES
CoRM21104	GGNNNUAUUCC	((.......))	4.12E-05	4.12E-05	NO
CoRM21126	ACAGGCNNGU	((......))	4.13E-05	4.13E-05	NO
CoRM21293	NNNCNCAGCNNN	(((......)))	4.23E-05	4.89E-07	NO
CoRM21328	NUUGCNNNNNNNNNNNGYRRN	(((((((.......)))))))	4.24E-05	7.18E-05	YES
CoRM21454	YYRUNNNANAUGG	(((((...)))))	4.33E-05	8.12E-07	NO
CoRM21561	GRRNNNAAGUUC	(((......)))	4.39E-05	4.39E-05	NO
CoRM21745	NNGGUGCGNNNYYNN	((((.......))))	4.49E-05	0.000836763	YES
CoRM22145	NNNAANAANNNNUNUUNNN	(((((((.....)))))))	4.74E-05	0.0610512	YES

Supplementary Table 2 – Predicted motifs that are supported by analysis of previously published microarray/RNA-seq data or by microarray analysis of chemical perturbations reported in this study. The structure of a motif is shown only when the *p*-value of conservation is better with the structure compared to the sequence alone. Otherwise, the motif is shown as a linear sequence.

Motif	Sequence/structure	Conservation *p*-value (sequence and structure)	Conservation *p*-value (sequence only)	Notes
CoRM1	C-A-U-A-G-A-N	2.12×10^{-29}	2.12×10^{-29}	• Known binding site for cycling sequence-binding proteins<br>• Up-regulated in stationary-phase PF<br>• Co-regulated across different experiments
CoRM13	U-N-A-U-G-G-A	1.43×10^{-19}	1.43×10^{-19}	• Down-regulated in stationary-phase PF<br>• Co-regulated across different experiments
CoRM15	U-Y-G-A-N-G-A	1.64×10^{-18}	1.64×10^{-18}	• Up-regulated in stumpy BF<br>• Down-regulated in EtBr-treated PF<br>• Up-regulated in HCl-treated PF<br>• Co-regulated across different experiments
CoRM17	A-U-G-U-A-N	3.65×10^{-18}	3.65×10^{-18}	• Down-regulated in stumpy BF<br>• Up-regulated in stationary-phase PF<br>• Up-regulated in PARN1-overexpressing PF<br>• Up-regulated in EtBr-treated PF<br>• Down-regulated in HCl-treated PF<br>• Down-regulated in verapamil-treated PF<br>• Co-regulated across different experiments<br>• The expression profiles of several PUF proteins show correlation or anti-correlation with the expression profiles of CoRM17-containing transcripts, suggesting RNA-stabilizing or RNA-destabilizing roles for these proteins.
CoRM23	U-Y-G-C-N-G-A	1.06×10^{-17}	1.06×10^{-17}	• Up-regulated in slender BF<br>• Down-regulated in stationary-phase PF<br>• Down-regulated in PARN1-overexpressing PF<br>• Down-regulated in EtBr-treated PF<br>• Up-regulated in HCl-treated PF<br>• Co-regulated across different experiments
CoRM25	U-Y-C-G-N-G-A	3.95×10^{-17}	3.95×10^{-17}	• Up-regulated in HCl-treated PF<br>• Co-regulated across different experiments
CoRM39	G-C-N-C-C-N-N-N-N-G-Y	5.02×10^{-16}	5.02×10^{-16}	• Down-regulated in stationary-phase PF<br>• Down-regulated in PARN1-overexpressing PF<br>• Down-regulated in DHH1-overexpressing PF<br>• Down-regulated in EtBr-treated PF<br>• Up-regulated in DMSO-treated PF<br>• Up-regulated in HCl-treated PF<br>• Down-regulated in hygromycin-treated PF<br>• Co-regulated across different experiments

CoRM80	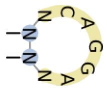	9.42×10^{-14}	9.54×10^{-07}	• Down-regulated in PARN1-overexpressing PF<br>• Down-regulated in EtBr-treated PF<br>• Up-regulated in HCl-treated PF<br>• Co-regulated across different experiments
CoRM127	C-N-A-A-G-G-N-N-G	1.24×10^{-12}	1.24×10^{-12}	• Down-regulated in DHH1-overexpressing PF<br>• Down-regulated in mutant DHH1-expressing PF<br>• Up-regulated in NaOH-treated PF<br>• Co-regulated across different experiments
CoRM176	U-G-G-C-C-N-N-N-Y-Y-R	4.81×10^{-12}	4.81×10^{-12}	• Co-regulated across different experiments
CoRM234	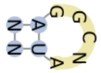	2.12×10^{-11}	6.33×10^{-09}	• Down-regulated in *in vitro*-cultured BF<br>• Co-regulated across different experiments
CoRM360	G-G-U-N-N-A-N-U-A-C-C	1.07×10^{-10}	1.07×10^{-10}	• Down-regulated in stationary-phase PF<br>• Co-regulated across different experiments
CoRM509	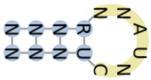	3.53×10^{-10}	0.002543	• Co-regulated across different experiments
CoRM580	A-C-U-G-G-A-R-G-U	5.46×10^{-10}	5.46×10^{-10}	• Down-regulated in stationary-phase PF
CoRM1374	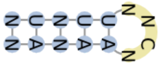	9.19×10^{-09}	0.000425	• Up-regulated in DHH1-overexpressing PF
CoRM1970	A-U-U-U-A-U-U	2.86×10^{-08}	2.86×10^{-08}	• Down-regulated in stumpy and slender BF<br>• Up-regulated in stationary-phase PF<br>• Up-regulated in PARN1-overexpressing PF<br>• Up-regulated in DHH1-overexpressing PF<br>• Up-regulated in EtBr-treated PF<br>• Down-regulated in DMSO-treated PF<br>• Down-regulated in HCl-treated PF<br>• Down-regulated in verapamil-treated PF<br>• Co-regulated across different experiments<br>• Three ELAV-like proteins Tb927.3.2930 (*Tb*RBP6), Tb927.7.5380 and Tb927.8.6650 bind to CoRM1970. The former two RBPs destabilize their ARE targets, while the latter stabilizes them, as suggested by inhibition and phenotypic activation of each protein.<br>• The expression of exosome exonuclease RRP45 is anti-correlated with the expression CoRM1970-containing transcripts, suggesting an ARE-mediated RNA-destabilizing role for RRP45.
CoRM2405	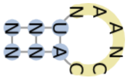	5.29×10^{-08}	2.05×10^{-05}	• Down-regulated in slender vs. stumpy BF

CoRM2447	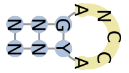	5.58×10^{-08}	0.194717	• Down-regulated in hygromycin-treated PF
CoRM3907	Y U U-Y-R-N-N-A-U-G-A-A-G	2.46×10^{-07}	2.46×10^{-07}	• Down-regulated in stationary-phase PF<br>• Up-regulated in mutant DHH1-expressing PF<br>• Down-regulated in DMSO-treated PF<br>• Co-regulated across different experiments
CoRM3981	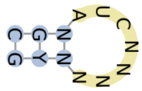	2.61×10^{-07}	8.76×10^{-06}	• Co-regulated across different experiments
CoRM4905	Y-U-G-Y-R-N-N-C-U-G-C-A-G	4.97×10^{-07}	4.97×10^{-07}	• Up-regulated in verapamil-treated PF
CoRM5069	C-A-G-G-G-N-N-N-Y-U-G	5.53×10^{-07}	5.53×10^{-07}	• Co-regulated across different experiments
CoRM6152	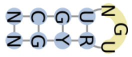	9.82×10^{-07}	2.96×10^{-05}	• Up-regulated in verapamil-treated PF
CoRM7127	A-G-U-C-N-G-N-N-N-N-R-Y-U	1.53×10^{-06}	1.53×10^{-06}	• Co-regulated across different experiments
CoRM7200	G-N-C-U-C-N-A-C	1.57×10^{-06}	1.57×10^{-06}	• Up-regulated in HCl-treated PF
CoRM7840	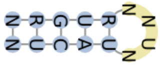	2.06×10^{-06}	6.20×10^{-05}	• Down-regulated in slender vs. stumpy BF<br>• Co-regulated across different experiments
CoRM9676	Y-U-G-A-G-C-A-G	3.93×10^{-06}	3.93×10^{-06}	• Co-regulated across different experiments
CoRM9692	U-U-C-G-C-C-N-G-R-R	3.95×10^{-06}	3.95×10^{-06}	• Co-regulated across different experiments
CoRM9755	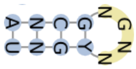	4.01×10^{-06}	0.000154	• Down-regulated in Alba3/4 RNAi in PF<br>• Co-regulated across different experiments
CoRM9912	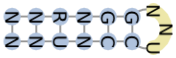	4.28×10^{-06}	0.194017	• Down-regulated in DHH1-overexpressing PF
CoRM12631	Y-N-G-N-N-N-U-G-C-C-N-G	9.10×10^{-06}	3.45×10^{-08}	• Down-regulated in stationary-phase PF
CoRM15495	G-Y-R-R-N-C-U-U-U-G-C	1.68×10^{-05}	1.68×10^{-05}	• Up-regulated in slender BF<br>• Co-regulated across different experiments
CoRM20879	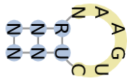	3.98×10^{-05}	0.033252	• Up-regulated in Alba3/4 RNAi in PF

CoRM21328	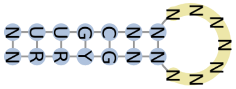	4.24×10^{-05}	7.18×10^{-05}	• Up-regulated in NaOH-treated PF
CoRM22145	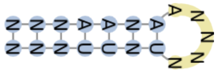	4.74×10^{-05}	0.061051	• Up-regulated in stationary-phase PF<br>• Co-regulated across different experiments

Supplementary Table 3 – Conserved motifs with reciprocal best matches among binding preferences of trypanosomatid RNA-binding proteins, as determined by RNAcompete assays (18). For identification of matching motifs, the RNAcompete probes were scanned for each of the 388 conserved trypanosomatid motifs, and probes with matches to each of the motifs were identified. The enrichment of each motif among protein-bound probes in each RNAcompete assay was examined using Mann-Whitney U test of ranks, and significant enrichments were identified (Benjamini correction, FDR < 0.05). A motif-protein pair was deemed to represent a reciprocal best match if that particular motif had the highest enrichment score for that protein and that protein had the highest enrichment score for that motif.

Motif	Sequence	Best matching RNAcompete assay	Enrichment z-score (p-value)	Protein used in the assay	*T. brucei* ortholog(s)	Validated by expression analysis
CoRM1	CAUAGAN	RNCMPT00216	16.7 (3.9×10^{-63})	Tbg972.3.4300	Tb927.3.3930	Y
CoRM15	UYGANGA	RNCMPT00254	19.2 (2.6×10^{-82})	LmjF35.2550	Tb09.211.4120	Y
CoRM17	AUGUAN	RNCMPT00217	32.2 (2.5×10^{-227})	Tbg972.6.2300	Tb927.6.2550	Y
CoRM39	GCNCCNNNNGY	RNCMPT00251	5.3 (4.5×10^{-8})	Tbg972.11.1000	Tb11.03.0170	Y
CoRM1970	AUUUAUU	RNCMPT00219	10.3 (2.3×10^{-25})	Tbg972.8.6820	Tb927.8.6650	Y
CoRM2871	CAAAAC	RNCMPT00255	25.3 (2.6×10^{-141})	LmjF35.4130	Tb09.211.0930, Tb09.211.2150	N
CoRM7200	GNCUCNAC	RNCMPT00220	9.4 (2.0×10^{-21})	Tbg972.9.4840	Tb09.211.0560, Tb11.01.5690	Y

Supplementary Table 4 – Primers used to amplify genes and gene fragments from genomic DNA of *T. brucei* 29-13, followed by cloning into different vectors for bacterial expression (pET-30b), RNAi (p2T7), or over-expression in *T. brucei* (pLew79).

Construct	Forward primer	Reverse primer
pET30b-RBP6	5′-GCCCAGATCTGGATCTACCCTCGCTGGAAAG-3′	5′-GGTGCTCGAGACCTACACGCATGGTGGGAG-3′
p2T7-RBP6	5′-ACACGGATCCCAACAGCCGTATCATCCCTT-3′	5′-ACACAAGCTTACTCATTCGCTCCACAGCTTC-3′
p2T7-DRBD12	5′-ACACGGATCCACAAGTTTGAATCATCCGCC-3′	5′-ACACAAGCTTAACCGTTTTCATCGGACAAGG-3′
p2T7-DRBD13	5′-ACACGGATCCGCAACCTCTTCATATCGGGA-3′	5′-ACACAAGCTTCGCCTGCTTACCAGAAGAAC-3′
pLew79-RBP6	5′-ACACAAGCTTATGTTCTACCCCAACAGCCC-3′	5′-ATATGGATCCACCAGCGGCACCGCGG-3′
pLew79-DRBD12	5′-GCGCAAGCTTATGAGCTTATTTTCTTTTACGCC-3′	5′-ATATGGATCCCGTAAGTGCAGCAAATGTCCC-3′
pLew79-DRBD13	5′-ACACAAGCTTATGACAGACCACCAAGTCATCTG-3′	5′-ATATGGATCCACCTACACGCATGGTGGGAG-3′

References

1. Aslett, M., Aurrecoechea, C., Berriman, M., Brestelli, J., Brunk, B.P., Carrington, M., Depledge, D.P., Fischer, S., Gajria, B., Gao, X. *et al.* (2010) TriTrypDB: a functional genomic resource for the Trypanosomatidae. *Nucleic Acids Res*, **38**, D457-462.
2. Chen, F., Mackey, A.J., Stoeckert, C.J., Jr. and Roos, D.S. (2006) OrthoMCL-DB: querying a comprehensive multi-species collection of ortholog groups. *Nucleic Acids Res*, **34**, D363-368.
3. Kramer, S., Queiroz, R., Ellis, L., Hoheisel, J.D., Clayton, C. and Carrington, M. (2010) The RNA helicase DHH1 is central to the correct expression of many developmentally regulated mRNAs in trypanosomes. *J Cell Sci*, **123**, 699-711.
4. Kabani, S., Fenn, K., Ross, A., Ivens, A., Smith, T.K., Ghazal, P. and Matthews, K. (2009) Genome-wide expression profiling of in vivo-derived bloodstream parasite stages and dynamic analysis of mRNA alterations during synchronous differentiation in Trypanosoma brucei. *BMC Genomics*, **10**, 427.
5. Jensen, B.C., Sivam, D., Kifer, C.T., Myler, P.J. and Parsons, M. (2009) Widespread variation in transcript abundance within and across developmental stages of Trypanosoma brucei. *BMC Genomics*, **10**, 482.
6. Veitch, N.J., Johnson, P.C., Trivedi, U., Terry, S., Wildridge, D. and MacLeod, A. (2010) Digital gene expression analysis of two life cycle stages of the human-infective parasite, Trypanosoma brucei gambiense reveals differentially expressed clusters of co-regulated genes. *BMC Genomics*, **11**, 124.
7. Utter, C.J., Garcia, S.A., Milone, J. and Bellofatto, V. (2011) Poly(A)-specific Ribonuclease (PARN-1) function in stage-specific mRNA turnover in Trypanosoma brucei. *Eukaryot Cell*.
8. Nilsson, D., Gunasekera, K., Mani, J., Osteras, M., Farinelli, L., Baerlocher, L., Roditi, I. and Ochsenreiter, T. (2010) Spliced leader trapping reveals widespread alternative splicing patterns in the highly dynamic transcriptome of Trypanosoma brucei. *PLoS Pathog*, **6**.
9. Haanstra, J.R., Kerkhoven, E.J., van Tuijl, A., Blits, M., Wurst, M., van Nuland, R., Albert, M.A., Michels, P.A., Bouwman, J., Clayton, C. *et al.* (2011) A domino effect in drug action: from metabolic assault towards parasite differentiation. *Mol Microbiol*, **79**, 94-108.
10. Shateri Najafabadi, H. and Salavati, R. (2010) Functional genome annotation by combined analysis across microarray studies of Trypanosoma brucei. *PLoS Negl Trop Dis*, **4**.
11. Holzer, T.R., McMaster, W.R. and Forney, J.D. (2006) Expression profiling by whole-genome interspecies microarray hybridization reveals differential gene expression in procyclic promastigotes, lesion-derived amastigotes, and axenic amastigotes in Leishmania mexicana. *Mol Biochem Parasitol*, **146**, 198-218.
12. Ubeda, J.M., Legare, D., Raymond, F., Ouameur, A.A., Boisvert, S., Rigault, P., Corbeil, J., Tremblay, M.J., Olivier, M., Papadopoulou, B. *et al.* (2008) Modulation of gene expression in drug resistant Leishmania is associated with gene amplification, gene deletion and chromosome aneuploidy. *Genome Biol*, **9**, R115.
13. Rochette, A., Raymond, F., Ubeda, J.M., Smith, M., Messier, N., Boisvert, S., Rigault, P., Corbeil, J., Ouellette, M. and Papadopoulou, B. (2008) Genome-wide gene expression profiling analysis of Leishmania major and Leishmania infantum developmental stages reveals substantial differences between the two species. *BMC Genomics*, **9**, 255.

14. Stuart, K., Gobright, E., Jenni, L., Milhausen, M., Thomashow, L. and Agabian, N. (1984) The IsTaR 1 serodeme of Trypanosoma brucei: development of a new serodeme. *J Parasitol*, **70**, 747-754.
15. Queiroz, R., Benz, C., Fellenberg, K., Hoheisel, J.D. and Clayton, C. (2009) Transcriptome analysis of differentiating trypanosomes reveals the existence of multiple post-transcriptional regulons. *BMC Genomics*, **10**, 495.
16. Panigrahi, A.K., Schnaufer, A., Ernst, N.L., Wang, B., Carmean, N., Salavati, R. and Stuart, K. (2003) Identification of novel components of Trypanosoma brucei editosomes. *Rna*, **9**, 484-492.
17. Redmond, S., Vadivelu, J. and Field, M.C. (2003) RNAit: an automated web-based tool for the selection of RNAi targets in Trypanosoma brucei. *Mol Biochem Parasitol*, **128**, 115-118.
18. Ray, D., Kazan, H., Cook, K.B., Weirauch, M.T., Najafabadi, H.S., Li, X., Gueroussov, S., Albu, M., Zheng, H., Yang, A. *et al.* (2013) A compendium of RNA binding motifs for decoding gene regulation. *Nature*, **In Press**.
19. Ray, D., Kazan, H., Chan, E.T., Pena Castillo, L., Chaudhry, S., Talukder, S., Blencowe, B.J., Morris, Q. and Hughes, T.R. (2009) Rapid and systematic analysis of the RNA recognition specificities of RNA-binding proteins. *Nat Biotechnol*, **27**, 667-670.
